# Three-dimensional open nano-netcage electrocatalysts for efficient pH-universal overall water splitting

**DOI:** 10.1038/s41467-019-12885-0

**Published:** 2019-10-25

**Authors:** Zewen Zhuang, Yu Wang, Cong-Qiao Xu, Shoujie Liu, Chen Chen, Qing Peng, Zhongbin Zhuang, Hai Xiao, Yuan Pan, Siqi Lu, Rong Yu, Weng-Chon Cheong, Xing Cao, Konglin Wu, Kaian Sun, Yu Wang, Dingsheng Wang, Jun Li, Yadong Li

**Affiliations:** 1 Department of Chemistry, Tsinghua University, Beijing, 100084 China; 2 Department of Chemistry, Southern University of Science and Technology, Shenzhen, 518055 China; 3 State Key Lab of Organic-Inorganic Composites and College of Chemical Engineering, Beijing University of Chemical Technology, Beijing, 100029 China; 4 Beijing National Center for Electron Microscopy and Laboratory of Advanced Materials, Department of Materials Science and Engineering, Tsinghua University, Beijing, 100084 China; 5 Shanghai Synchrotron Radiation Facilities, Shanghai Institute of Applied Physics, Chinese Academy of Science, Shanghai, 201800 China

**Keywords:** Energy, Electrocatalysis, Nanoscale materials

## Abstract

High-efficiency water electrolysis is the key to sustainable energy. Here we report a highly active and durable RuIrO_*x*_ (*x* ≥ 0) nano-netcage catalyst formed during electrochemical testing by *in-situ* etching to remove amphoteric ZnO from RuIrZnO_*x*_ hollow nanobox. The dispersing-etching-holing strategy endowed the porous nano-netcage with a high exposure of active sites as well as a three-dimensional accessibility for substrate molecules, thereby drastically boosting the electrochemical surface area (ECSA). The nano-netcage catalyst achieved not only ultralow overpotentials at 10 mA cm^−2^ for hydrogen evolution reaction (HER; 12 mV, pH = 0; 13 mV, pH = 14), but also high-performance overall water electrolysis over a broad pH range (0 ~ 14), with a potential of mere 1.45 V (pH = 0) or 1.47 V (pH = 14) at 10 mA cm^−2^. With this universal applicability of our electrocatalyst, a variety of readily available electrolytes (even including waste water and sea water) could potentially be directly used for hydrogen production.

## Abstract (web summary)

Water electrolysis is considered a key reaction for future sustainable fuel generation. Here, authors report a three-dimensional RuIrOx nano-netcage catalyst that shows high activities and efficiencies for pH-universal overall water splitting.

## Introduction

An important approach to a sustainable energy future is via the energy conversions based on “electrochemical water cycle”, that is, electrolyzing water for H_2_, which is subsequently fed to fuel cells for electricity^1–4^. Currently, the ever-decreasing electricity price (average 6.71 cents/kwh for industrial electricity in the United States on May 2019, U.S. Energy Information Administration) is stimulating extensive research efforts on the exploration of advanced catalysts for water electrolysis, and the overpotentials of hydrogen and oxygen evolution half reactions (HER and OER) have been continually lowered^5–9^. However, most of the reported catalysts were aiming at only HER or OER half reaction under a particular electrolyte condition (pH = 0 or 14)^5–9^. Whereas in practical applications, it is highly desirable to develop a high-performance catalyst that is universally compatible (pH-universal, and bi-functional for both cathode and anode), so as to enable all kinds of electrolytes (even including waste water and sea water) to be directly used for hydrogen production, and to optimize the reliability during operation^10–13^. Moreover, such a universal catalyst could work regardless of the pH value of electrolyte and without the necessity of distinguishing the cathode and anode, thereby offering new opportunities for exploring next-generation water splitting technologies.

For supported electrocatalysts, a commonly less noticed fact is that the catalytic particles are often partially (even mostly) embedded in the support material, rendering part of the catalytic surface ineffective; and the ratio of ineffective surface increases with particle size decreasing^14–16^. Alternatively, a higher atomic utilization could be achieved by designing open three-dimensional nanostructures^17,18^. There have been several studies reporting the nanoscale hollow porous structure (such as boxes and cages) as good electrocatalysts^19–21^. Among them, the metal-organic framework (MOF) has attracted great interests as a precursor to hollow nanostructure^22–27^. However, most of these materials were stacked by nanoparticles, which still have considerable limitations on the exposure of active sites exposure and the diffusion of substrate molecules^23–27^.

**Fig. 1:**
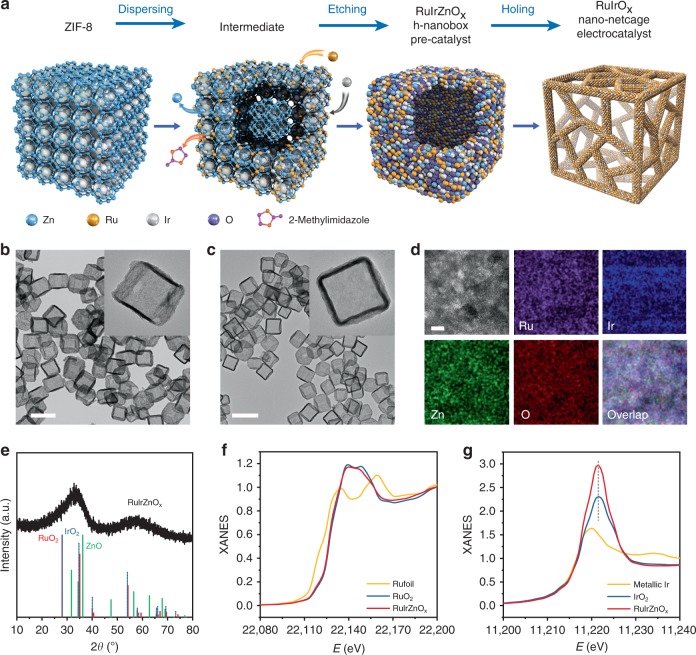
Synthetic scheme and Characterization of RuIrZnO_*x*_ *h-*nanoboxes. **a** Schematic illustration of the synthetic process. **b** TEM and magnified (inset) images of RuIrZnO_*x*_-U nanoboxes. Scale bar: 200 nm. **c** TEM and magnified (inset) images of RuZnO_*x*_-U nanoboxes. Scale bar: 200 nm. **d**, **e** AC HAADF-STEM image (scale bar: 2 nm) and atomic-resolution EDX elemental mapping (**d**), XRD pattern (**e**) of RuIrZnO_*x*_ *h-*nanoboxes. **f**, **g** Normalized XANES spectra of RuIrZnO_*x*_ *h*-nanoboxes at Ru K-edge and Ir L_3_-edge

Here we developed a Dispersing-Etching-Holing (DEH) strategy to prepare the high-performance catalyst (Fig. 1a). Using this DEH strategy, we successfully obtained nanosized hollow RuIrO_*x*_ (*x* ≥ 0) nano-netcages with their surfaces three-dimensionally accessible for substrate molecules. The open nano-netcage features six mesh-like walls made of interconnecting ultrathin nanowires, with abundant mesoporous structures, which in combination remarkably enhance the atom utilization of Ru/Ir. Further investigation revealed that the nano-netcage were ca. five times and ca. three times in electrochemical active surface area (ECSA) with respect to particulate commercial Ru/C and Ir/C catalysts. The robust secondary hollow architecture self-supported by nanowires guarantees a high durability after multiple electrochemical test cycles. Density functional theory (DFT) investigation and the operando X-ray absorption spectroscopy (XAS) studies reveal that the introduction of Ir into RuO_*x*_ remarkably decreases the energy barriers for both HER and OER; and prevents Ru from over-oxidation under acidic OER conditions, therefore significantly alleviates the long-suffering OER stability problems of precious metal catalysts.

## Results

### Synthesis strategy and characterization of RuIrZnO_*x*_ hollow nanoboxes

The DEH strategy starts with evenly (ideally at the atomic level) dispersing the catalytically active components into an etchable parent material. Zeolitic imidazolate framework (ZIF-8) is an ideal precursor for dispersing Ru/Ir components, because the Zn^2+^ in its skeleton can readily exchange with Ru^3+^/Ir^4+^, and the volume of a single cavity can accommodate 1–2 hydrated Ru^3+^/Ir^4+^ ions^28–32^. During solvothermal reaction, the Ru^3+^/Ir^4+^/Zn^2+^ ions undergo hydrolysis, and the internal ZIF-8 core are gradually etched, yielding hollow nanoboxes which are further annealed to obtain the homogeneous composite oxides. Subsequently, the amphoteric ZnO is removed by electrochemical in situ etching, and the walls of nanoboxes are holed at the same time, leaving the final nano-netcages (Fig. 1a).

In a typical experiment, RuCl_3_·*x*H_2_O (13.0 mg) and Na_2_IrCl_6_·6H_2_O (40.5 mg) were added to a water/methanol mixed dispersion of ZIF-8 nanocubes (70–100 nm in edge length, Supplementary Fig. 1). After solvothermal treatment (80 °C for 2 h), the RuIrZnO_*x*_-U (U stands for unannealed) were obtained (Fig. 1b, Supplementary Fig. 2). RuIrZnO_*x*_-U were further annealed at 350 °C for 2 h under 0.5% O_2_/N_2_ atmosphere to obtain the RuIrZnO_*x*_ hollow nanoboxes (*h*-nanoboxes). Similarly, RuZnO_*x*_ *h*-nanoboxes and the samples with different Ru/Ir ratios were obtained by regulating the introduction amount of Ir^4+^ (Fig. 1c, Supplementary Figs. 3–5).

As shown in the transmission electron microscopy (TEM) and scanning electron microscopy (SEM) images (Supplementary Figs. 6, 7), the RuIrZnO_*x*_ *h*-nanoboxes inherited the cubic shape (70–100 nm in edge length), with a hollow interior enclosed by thin walls (ca. 6.0 nm in thickness). Brunauer–Emmett–Teller (BET) adsorption–desorption isotherms indicated that the RuIrZnO_*x*_ *h*-nanoboxes exhibited a high surface area of 78.32 m^2^ g^−1^ (Supplementary Fig. 8). A poor crystallinity was evidenced by the broadened X-ray diffraction (XRD) pattern (Fig. 1e). The Ru:Ir:Zn molar ratio was 1:0.47:1.07 (Supplementary Table 1, determined by inductively coupled plasma-optical emission spectrometry, ICP-OES). X-ray absorption near-edge structure (XANES) shows that the absorption threshold position of Ru and the white line position of Ir for RuIrZnO_*x*_ were close to that of RuO_2_ and IrO_2_, respectively, implying the similar oxidation state (Fig. 1f, g). Extended X-ray absorption fine structure (EXAFS) shows that both Ru and Ir in RuIrZnO_*x*_ are coordinated to O, indicating the oxides nature of RuIrZnO_*x*_ (Supplementary Fig. 9). The Raman spectrum, X-ray photoelectron spectroscopy (XPS), thermogravimetric analysis (TGA) and Fourier-transform infrared spectroscopy (FT-IR) further confirmed the oxide characteristic of RuIrZnO_*x*_ *h*-nanoboxes (Supplementary Figs. 9 and 10). High-resolution transmission electron microscopy (HRTEM) characterization on an individual RuIrZnO_*x*_ *h*-nanobox revealed no obvious lattice fringe on the wall, and SAED pattern presented a typical ring of amorphism (Supplementary Fig. 11). The aberration-corrected high-angle annular dark-field scanning transmission electron microscope (AC HAADF-STEM) image of the outer wall showed that the metal atoms displayed a random and even distribution (Supplementary Fig. 11). The uniform blend of Ru/Ir/Zn also can be confirmed by the atomic-resolution energy-dispersive X-ray spectroscopy (EDX) element mapping (Fig. 1d). To sum up, it can be identified that the RuIrZnO_*x*_ *h*-nanoboxes contained composite oxides of Ru/Ir/Zn, with the three metal ions mixed at the atomic level, resulting in a distinct amorphous feature. RuZnO_*x*_ *h*-nanoboxes also showed analogous morphology and structural features (Supplementary Fig. 12).

### HER and OER performances

The RuIrZnO_*x*_ *h*-nanoboxes are endowed with multiple functionalities: (1) ZnO could be in situ etched at acidic or alkaline electrochemical conditions to form RuIrO_*x*_, rendering the active Ru/Ir components highly exposed, and leading to, hopefully, a drastic elevation in electrocatalytic activity; (2) under the HER operating potential, Ru/Ir oxides could be in situ reduced to elemental Ru/Ir, both possessing a proper metal-H* binding energy, promising an excellent HER activity^14,33,34^; (3) Ru/Ir oxides are already known as good OER catalysts, so the RuIrO_*x*_ can potentially act as anode catalyst in water electrolysis^35,36^. Therefore, the RuIrZnO_*x*_ *h*-nanoboxes are expected to work as pre-catalyst for in situ generating both high-performance HER and OER electrocatalysts. We then assessed the HER and OER performances of the in situ generated RuIrO_*x*_ catalyst in acidic and alkaline conditions.

**Fig. 2:**
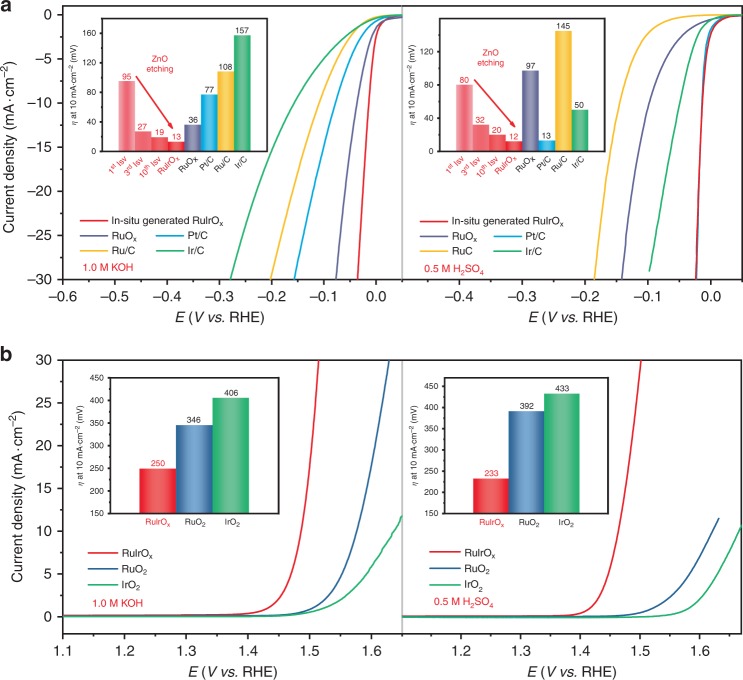
Electrochemical properties of the in situ generated RuIrO_*x*_ catalyst. HER (**a**) and OER (**b**) polarization curves and the overpotentials at 10 mA cm^−2^ of the electrocatalysts (insets) in 1.0 M KOH solution (left) and 0.5 M H_2_SO_4_ solution (right). Scan rate: 1 mV s^−1^

As shown in Fig. 2a, in 1.0 M aqueous KOH solution, freshly prepared RuIrZnO_*x*_ *h*-nanoboxes exhibited a HER overpotential of 95 mV (IR corrected) versus reversible hydrogen electrode (RHE, Supplementary Fig. 13) to deliver a current density of 10 mA cm^−2^. As the in situ etching of ZnO progressed, the HER overpotential gradually lowered, and finally stabilized at 13 mV (Supplementary Figs. 14 and 15), which is the lowest overpotential ever reported so far to the best of our knowledge (Supplementary Table 2). The HER overpotential at 10 mA cm^−2^ (13 mV) and the resulting Tafel slope (23 mV dec^−1^) of in situ generated RuIrO_*x*_ catalyst are both significantly lower than that of Ru/C, Ir/C, Pt/C, and RuO_*x*_ (Fig. 2a, Supplementary Fig. 16), indicating a most efficient HER kinetics. In 0.5 M H_2_SO_4_ solution, as the etching of ZnO progressed, the HER overpotential at 10 mA cm^−2^ of the electrocatalyst decreased from an initial 80 mV to a final 12 mV (Supplementary Fig. 15), which is comparable to that of commercial Pt/C (13 mV), and far lower than that of Ru/C, RuO_*x*_, and Ir/C (Fig. 2a, Supplementary Table 3). The in situ generated RuIrO_*x*_ catalyst was tested for 3000 cyclic voltammetry (CV) cycles in acidic/alkaline medium, and in neither case did the HER activity show any noticeable change, demonstrating an excellent stability for long-term electrochemical process (Supplementary Fig. 17).

For OER, in 0.5 M H_2_SO_4_ solution, the in situ generated RuIrO_*x*_ catalyst gave an OER overpotential of 233 mV at 10 mA cm^−2^ (Fig. 2b) and a Tafel slope of 42 mV dec^−1^ (Supplementary Fig. 18), both significantly lower than that of commercial RuO_2_ (392 mV, 65 mV dec^−1^) and IrO_2_ (433 mV, 70 mV dec^−1^). In electrolyte of KOH (1.0 M) at 10 mA cm^−2^, the RuIrO_*x*_ catalyst also exhibited the lowest OER overpotential (250 mV) and Tafel slope (50 mV dec^−1^) among the three catalysts. Ru-based catalysts generally display high activities under acidic OER conditions, but suffer from low stability^37–39^. For our catalyst here, the introduction of Ir component remarkably improved the stability, with no obvious change in activity after 3000 CV cycles in acidic condition. In contrast, the Ir-free RuO_*x*_ catalyst displayed a severe decline in activity after only five cycles (Supplementary Figs. 19 and 20).

It is worth mentioning that the acidic polymer electrolyte membrane (PEM) water electrolyzer has become the most promising alternative for H_2_ production by virtue of its high efficiency, large operating pressure and exceptionally long, maintenance-free life^40,41^. Therefore, noble-metal-based catalysts are still playing an irreplaceable role in OER owing to their good resistance to acid compared with non-precious materials. Our RuIrO_*x*_ nano-netcages display not only high HER/OER activities in acidic condition, but also an excellent robustness against acid. Such a catalyst is expected to find practical applications in the PEM water electrolyzer.

### Characterization of in situ generated RuIrO_*x*_ nano-netcages and ECSA analysis

**Fig. 3:**
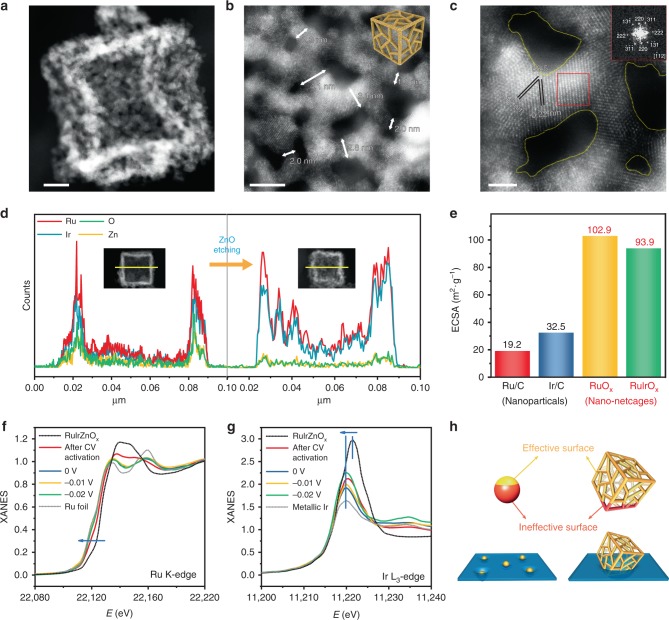
Structure analysis and the corresponding ECSA of the in situ generated RuIrO_*x*_ nano-netcages. **a–c** AC HAADF-STEM and magnified images of RuIrO_*x*_ nano-netcages. Scale bar: 10, 5, and 2 nm. The inset of (**c**) shows the corresponding FFT image of the selected region. **d** EDX spectroscopy line-scan profiles of the RuIrZnO_*x*_ and the in situ generated RuIrO_*x*_ nano-netcage. **e** The ECSA of the RuIrO_*x*_, RuO_*x*_ nano-netcages, commercial Ru/C, and Ir/C. **f**, **g** Normalized XANES spectra of RuIrO_*x*_ measured at different electrode potentials at Ru K-edge (**f**) and Ir L_3_-edge (**g**) during the HER process under alkaline condition. **h** Comparison of the ratios of effective surface for supported nanoparticles and the three-dimensional open nano-netcage structure

Further characterizations were conducted to unveil the underlying mechanism for the dramatic elevation in electrocatalytic activity after in situ etching ZnO. The in situ formed RuIrO_*x*_ were obtained after electrochemical activation (that is, running for several CV cycles within the potential range of specific electrochemical reactions). Here we use the case of HER in alkaline condition as an example (for other cases including OER, please refer to Supplementary Figs. 22–32 and Supplementary Note 1). The TEM image (Supplementary Fig. 21) shows that the walls of the in situ generated RuIrO_*x*_ have become rougher and more translucent, with the wall thickness decreased from 6 nm down to 2 nm. The AC HAADF-STEM image of an individual RuIrO_*x*_ cage is shown in Fig. 3a, and a magnified image in Fig. 3b. The walls after etching have become highly porous (1–4 nm in pore size) and mesh-like, collectively constructing a nano-netcage architecture self-supported by interconnecting ultrathin (2–4 nm in width) nanowires, which is distinctly different from the architectures for nanoboxes and nanoframes (Supplementary Fig. 25). The nano-netcage is mainly composed of Ru and Ir, with the X-ray counts of Zn and O are significantly decreased compared with the RuIrZnO_*x*_ before HER (Fig. 3d). It is worth emphasizing that the ZnO component could only be thoroughly removed via in situ electrochemical etching, which cannot be simply replaced by liquid-phase etching method^42^ (Supplementary Figs. 26 and 27). The XPS and ex situ XAS of RuIrO_*x*_ revealed that oxidation states of Ru and Ir decreased and the signals of metal-metal bonds emerged after HER activation, indicating the in situ reduction of Ru/Ir oxides (Supplementary Figs. 28–30). The atomic-resolution EDX elemental mapping (Supplementary Fig. 21) revealed a homogenous distribution of Ru/Ir, and the higher magnification AC HAADF-STEM image (Fig. 3c) indicates a crystalline characteristic of the RuIr alloy with a distinct cubic fast Fourier-transform (FFT) pattern. The high crystallinity of RuIr alloy, in combination with the innate stability of Ru/Ir noble metals, guarantees the structural integrity of the netcages.

The operando XAS experiments were further conducted to investigate the alteration in oxidation state of Ru and Ir during HER operation and to identify the real active sites. Under HER working potential (0 to −0.02 V), as shown in Fig. 3f, the absorption threshold of Ru K-edge shifted to lower energy, indicating a lowered oxidation state. EXAFS of Ru K-edge revealed that the peak attributed to Ru–O bond disappeared, and the peak at 2.35 Å (corresponding to Ru–Ru/Ir) becomes the main peak (Supplementary Fig. 31 and Supplementary Note 2). Likewise, the white line position of Ir L_3_-edge also shifted to lower energy, and the formal *d*-band hole count of Ir under HER potential was almost identical to that of metallic Ir, implying a similar oxidation state^43,44^ (Fig. 3g, Supplementary Fig. 31). The operando XAS results proved that, under HER operation, Ru/Ir oxides were almost completely reduced, and the afforded RuIr alloy served as the real active sites. The Ru:Ir molar ratio was determined as 2.01:1 by ICP-OES (Supplementary Table 4), which suggested that the in situ generated nano-netcage structure was Ru_2_Ir alloy.

As mentioned above, this three-dimensional open nano-netcage consists of interconnecting ultrathin nanowires of Ru_2_Ir alloy, which endows the catalyst with a high exposure of Ru/Ir active sites as well as a high accessibility for substrate molecules. This unique architecture can effectively circumvent the drawbacks of ultrafine particulate catalyst (for example, ca. 1.2 nm Ru and ca. 2.0 nm Ir nanoparticles of commercial Ru/C and Ir/C, Supplementary Fig. 34), including the activity loss owing to a large amount of surface active atoms embedded into the support, and the activity decline owing to particle aggregation during the operation^17,45^. We used the underpotential deposition of copper (Cu-UPD) (Supplementary Figs. 33–35) to determine the ECSA of the sample after etching, and found a fivefold and a threefold larger ECSA with respect to particulate Ru/C and Ir/C (Fig. 3e). According to the theoretical calculation results on specific surface area, the nano-netcage structure has the ratio of effective surface area around 70%, much higher than the particulate catalyst (3–24%), which indicates a significantly elevated exposure and atomic utilization of Ru/Ir in the nano-netcages than in supported Ru/C and Ir/C (Fig. 3h, Supplementary Note 3 and Supplementary Table 5). In the meantime, the unique open mesh-like texture could facilitate the diffusion and transfer of substrate molecules, thereby promoting the HER kinetics. Also, the steady hollow architecture guaranteed an excellent stability of the catalyst for 3000 CV tests.

In conclusion, this DEH strategy provides an efficient approach to elevating the utilization of catalytically active components (noble-metal atoms, in particular) and enhancing the performances of supported nanocatalysts.

### Theoretical insights on HER and OER activity and operando XAS study during acidic OER

**Fig. 4:**
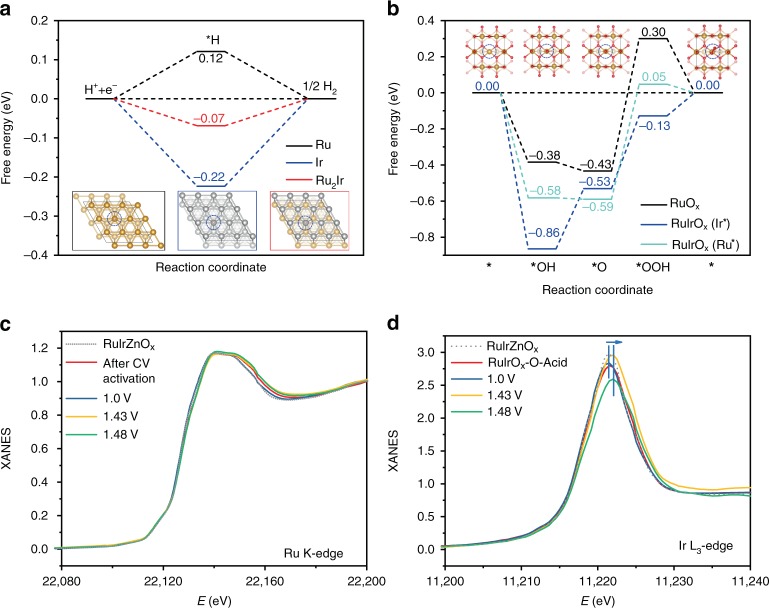
DFT calculation results and the operando XAS study during acidic OER. **a**, **b** DFT calculation of the predicted free energy profiles for HER on Ru (0001) (black line), Ir (111) (blue line) and Ru_2_Ir (111) (red line) surfaces at U = 0 eV (**a**) and the predicted free energy profiles for OER on RuO_2_ (110) surface (black line), RuIrO_*x*_ (110) surfaces with Ir (blue line) and Ru (cyan line) as the active sites at U = 1.23 eV (**b**). The H atoms are in pink, O in red, Ir in gray and Ru atoms in gold. **c**, **d** Normalized XANES spectra of RuIrO_*x*_ measured at different electrode potentials at Ru K-edge (**c**) and Ir L_3_-edge (**d**) during the OER process under acidic condition

Compared with Ir-free RuO_*x*_ nano-netcages, the RuIrO_*x*_ nano-netcages exhibited superior activities for both HER and OER. DFT calculations were further conducted to obtain in-depth understanding on the underlying mechanism of the excellent catalytic activity of RuIrO_*x*_ nano-netcage. The real active species for in situ generated RuIrO_*x*_ nano-netcages under HER situation has been proved to be Ru_2_Ir alloy as mentioned above. We determined the Ru_2_Ir (111) surface with the Ir–Ru–Ru layer-by-layer distributions as the most stable structure by calculating surface energies of three model surfaces (Supplementary Fig. 36). We confirmed that the Tafel step (2*H → H_2_ + 2*) is the rate determining step in both acidic and alkaline solutions (Supplementary Fig. 37 and Supplementary Note 4). Thus we considered the H adsorption as the key descriptor for HER, which was also suggested previously^46^ (Fig. 4a). The free energy profiles of Ru, Ir, and Ir-incorporated Ru_2_Ir systems indicate that the Ru_2_Ir presents the lowest energy barrier. Compared with pure Ru and Ir systems, apparent electron transfer from Ru to Ir atoms for Ru_2_Ir is revealed by the bader charges listed in Supplementary Table 6. Electron density difference (Supplementary Fig. 38) of Ru_2_Ir also shows electron accumulation at the Ir site, demonstrating the effectiveness of Ir incorporation for promoting the HER catalytic performance. We further considered the role of trace Zn. As can be seen from Supplementary Figs. 39 and 40, Zn atom at the Ru_2_Ir surface is not active for HER, and does not improve the catalytic activity. The incorporation of Zn into the slab of Ru_2_Ir does not help to improve the catalytic activity of Ir site as well.

We studied the reaction mechanisms to understand the performance of different ruthenium oxide (RuO_*x*_) catalysts for driving the OER. Previous work has proved that the oxygen coupling (*O + *O) reaction is less favorable than the formation of *OOH intermediate^47^. Tafel slopes in Supplementary Fig. 18 show that the rate determining step under both acidic and alkaline conditions is the third elementary step (*O → *OOH). Thus, the third step was studied in detail in Supplementary Figs. 41, 42 and Supplementary Note 5, which confirms the excellent OER activity of RuIrO_*x*_. The four-step OER mechanism was adopted by considering the stabilities of the intermediates. The predicted free energy profiles for OER catalyzed by pure RuO_*x*_ and Ir-incorporated RuIrO_*x*_ systems (Supplementary Fig. 43) are shown in Fig. 4b. We can see that the incorporation of Ir into ruthenium oxide tunes the intermediate adsorption energies and facilitates the reactions. The potential limiting step (PLS) for OER is the third electrochemical step from *O to *OOH, and the introduction of Ir remarkably decreases the energy barrier by this PLS. We can conclude that the stability of *OOH intermediate is the key descriptor of OER activity in this RuIrO_*x*_ system. Note that the Ir site is more favorable than the Ru site for OER, although the free energy profiles show synergy effect of Ir and Ru sites. And the Zn dopant does not contribute to the superior catalytic performance (Supplementary Figs. 44 and 45).

RuO_2_, though traditionally known as a good OER catalyst, suffers from lattice oxygen evolution reaction (LOER), and can be over-oxidized to form inactive RuO_4_, leading to a drastic decline in OER activity^48,49^. While in this work, by operando XAS characterization, we found that with the working potentials scanned from 1.0 to 1.48 V, the XANES spectra of Ru K-edge were essentially identical, and the position of Ru K-edge absorption threshold were barely changed (Fig. 4c, Supplementary Fig. 46), confirming the stability of the oxidation state of Ru. These results indicate that the introduction of Ir can effectively inhibit the over-oxidation of Ru, and stabilize the Ru species at relatively low oxidation states, and therefore protect the OER activity from declining. On the contrary, the white line position of Ir L_3_-edge gradually shifted to the higher energy during OER process, with the increased *d*-band hole counts, implying an elevated oxidation state of Ir under high OER potentials^43,44^ (Fig. 4d, Supplementary Fig. 46). These results indicate that in our RuIrO_*x*_ catalyst, Ir species may transfer electrons to Ru, thereby preventing Ru from over-oxidation. In the meantime, such Ir species of elevated oxidation states have been proved to be conducive to the OER activity^44^. Therefore, the introduction of Ir could effectively engineer the electronic structure of Ru with better resistance to the over-oxidation under acidic OER conditions, and boost the stability of the catalyst.

To summarize, by the incorporation of Ir into the RuO_*x*_, we have not only further enhanced the activities of both HER and OER, but also greatly alleviated the long-suffering stability problems typically for precious metal catalysts under acidic OER condition.

### pH-universal overall water splitting performance

**Fig. 5:**
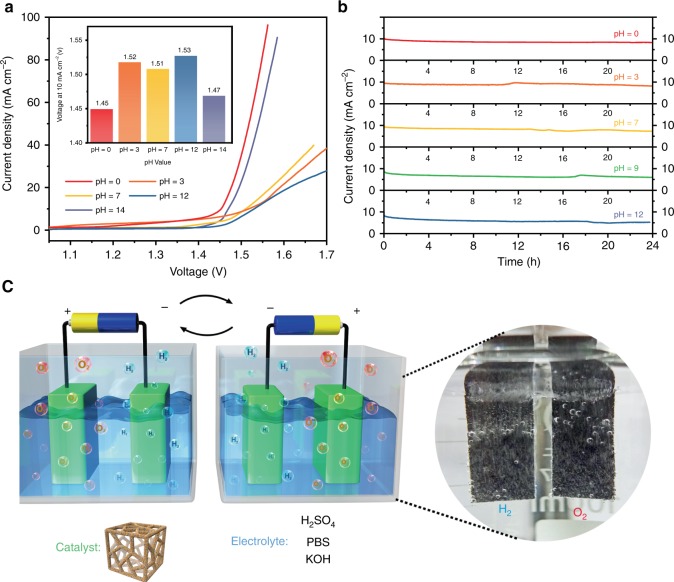
Overall water splitting performance of the RuIrO_*x*_ nano-netcages. **a** Polarization curves and the voltages at 10 mA·cm^2^ (inset) of the RuIrO_*x*_ nano-netcages over a broad pH range (0–14). **b** The current-time (*I*–*t*) curves of the RuIrO_*x*_ nano-netcages for 24 h at different pH values. **c** Schematic illustration of the water splitting setup with an AA battery showing that the RuIrO_*x*_ nano-netcages could work irrespective of the positive/negative terminals of the battery and the digital photograph of the evolution of H_2_ and O_2_ from the electrodes during electrolysis

The RuIrO_*x*_ nano-netcages are expected to work as a “universally compatible” electrocatalyst that simultaneously gives excellent HER and OER performances over the entire pH range. In the experiments of overall water splitting, the potential required for RuIrO_*x*_ nano-netcages was as low as 1.45 V at pH = 0 or 1.47 at pH = 14 (at 10 mA cm^−2^, Fig. 5a), both outperformed most of the reported electrocatalysts for water splitting (Supplementary Table 7); even at neutral pH, the required potential is only marginally higher (1.51 V at 10 mA cm^−2^), promising good operability, safety and environmental friendliness. The catalysts were durable for at least 24 h in electrolytes of various pH values, demonstrating a good stability (Fig. 5b). When powered merely by an AA battery (Supplementary Fig. 47), the RuIrO_*x*_ nano-netcages can catalyze water electrolysis over the entire pH range (0–14). Supplementary Movie 1 shows that large numbers of bubbles emerge at both electrodes immediately when power is switched on. This universally compatible catalyst transcends the limitation on the pH of electrolytes, and works irrespective of the positive/negative terminals of the battery (Fig. 5c). The excellent activity and good stability over a broad pH range (0–12) would hopefully enable the RuIrO_*x*_ nano-netcages to mitigate the main drawbacks of existing electrolysis technologies and provide possibilities of developing next-generation water splitting technologies.

In conclusion, we designed a three-dimensional open nano-netcage RuIrO_*x*_ electrocatalyst which maximized the atomic utilization of active noble metals. The RuIrO_*x*_ nano-netcages exhibit pH-universal (0–14) and high activity as well as stability for overall water splitting. This universality qualifies all kinds of aqueous solutions as available electrolytes (in particular, waste water and sea water), thereby promoting the technology of water electrolysis for sustainable energy toward real applications.

## Methods

### Chemicals

Analytical grade zinc nitrate hexahydrate (Zn (NO_3_)_2_·6H_2_O), iridium sodium hexachloroiridate hexahydtate (Na_2_IrCl_6_·6H_2_O) and commercial RuO_2_, IrO_2_, and Pt/C (20%) catalysts were purchased from Alfa Aesar. 2-methylimidazole was purchased from Aladdin. Ruthenium (III) chloride hydrate (RuCl_3_·*x*H_2_O) was purchased from SCRC. Commercial Ru/C (5%) electrocatalyst was purchased from Macklin. Commercial Ir/C (20%) electrocatalyst was purchased from Premetek. All chemicals were from commercial sources and used without further purification.

### Synthesis of ZIF-8

ZIF-8 was synthesized as previously reported after slight modifications^50^. Zn (NO_3_)_2_·_6_H_2_O (0.3626 g) was dissolved in 12.5 mL deionized water. 2-methylimidazole (5.6752 g) and CTAB (0.0175 g) were dissolved in 87.5 mL of deionized water. Then the former solution was rapidly injected into the latter followed by 5 min stirring for sufficient dispersion. The resulting suspension was allowed standing at 28 °C for 3 h to obtain ZIF-8 dispersed solution.

### Synthesis of RuIrZnO_*x*_

The as-obtained ZIF-8 suspension (8 mL) was centrifuged and washed with MeOH for several times. The precipitates were dispersed uniformly in 3 mL deionized water by sonication. RuCl_3_·*x*H_2_O (13 mg, 62.5 μmol) and Na_2_IrCl_6_·6H_2_O (40.5 mg, 72.5 μmol) were dissolved in 2 mL deionized water, respectively. Then, the RuCl_3_·*x*H_2_O, Na_2_IrCl_6_·6H_2_O solution and 1 mL MeOH were added into the ZIF-8 suspension under stirring for 5 min. The resulting suspension was transferred to 12 mL Teflon-lined stainless-steel autoclaves and then heated at 80 °C for 2 h. The precipitates were collected by centrifugation and washed with MeOH for several times, and dried at 60 °C. The obtained powders were annealed at 350 °C for 2 h under 0.5% O_2_/N_2_ atmosphere. The RuIrZnO_*x*_-25 and RuIrZnO_*x*_-50 were obtained when the introduction amount of Ir precursor were 25 and 50 μmol with constant amount of Ru precursor (62.5 μmol). The Ir-free RuZnO_*x*_ was obtained without the introduction of Ir precursor and the introduction amount of Ru precursor was 75 μmol.

### Characterization

Powder X-ray diffraction patterns (PXRD) of samples were recorded using a Bruker D8 Advance diffractometer (Cu Kα, *λ* = 1.5418 Å, operating at 40 kV/40 mA). The solid-state Raman spectra were recorded on a LabRAM HR Evolution microspectrophotometer (excitation wavelength, *λ* = 532 nm). The morphologies were characterized by transmission electron microscope (TEM, Hitachi-7700, 100 kV) and scanning electron microscope (SEM, Hitachi S-4800). The high-resolution transmission electron microscope (HRTEM), and the corresponding EDX line-scan profiles were carried out by a FEI Tecnai G2 F20 S-Twin HRTEM working at 200 kV. The aberration-corrected high-angle annular dark-field scanning transmission electron microscope (AC HAADF-STEM) images and the corresponding atomic-resolution energy-dispersive X-ray spectroscopy element mapping were recorded by a Titan 80-300 scanning/transmission electron microscope operated at 300 kV. N_2_ adsorption–desorption measurements were carried out at 77 K using a Quantachrome SI-MP Instrument. The surface area of the samples was estimated by method of Brunauer–Emmett–Teller (BET). Elemental analysis of Ru, Ir, and Zn in the samples was detected by a Thermo IRIS Intrepid II inductively coupled plasma-optical emission spectrometry (ICP-OES). X-ray photoelectron spectroscopy (XPS) experiments were performed on a ULVAC PHI Quantera microprobe. Thermogravimetric analysis (TGA) was carried out using a TGA Q5000 differential thermal analyzer. The samples were heated in air stream from room temperature to 500 °C with a heating rate of 10 °C/min. Fourier-transform infrared spectroscopy (FT-IR) spectra were recorded using a PE Spectrum I spectrophotometer in 4000–400 cm^−1^ region.

### Electrochemical measurements for HER and OER

All the electrochemical measurements were carried out with a three-electrode setup using a CHI 760E electrochemical workstation (CH Instruments, Inc., Shanghai). The synthesized RuIrZnO_*x*_ and RuZnO_*x*_ catalysts were dispersed onto carbon black (Vulcan XC72) with the noble metal/C ~20%. To prepare the working electrode, the catalysts (RuIrZnO_*x*_, RuZnO_*x*_ and commercial Pt/C, Ru/C, and Ir/C catalysts) were dispersed in 1 mL water/ethanol (*v*:*v*, 1:1) solution with 20 μL Nafion solution (5 wt%) by sonication to form a homogeneous ink. Then, the ink was loaded onto the surface of a rotating disk electrode (RDE) with a diameter of 2.5 mm, the precious metal loading of RuIrZnO_*x*_, RuZnO_*x*_, commercial Pt/C, Ru/C, and Ir/C catalysts was ~10 μg cm^−2^ (calculated based on ICP-OES). The HER performance was evaluated in N_2_ saturated 0.5 M H_2_SO_4_ and 1.0 M KOH solutions, the OER performance was evaluated in O_2_ saturated above-mentioned electrolyte. For HER and OER, the linear sweep voltammetry (LSV) was conducted with a rotation speed of 1600 r.p.m. The saturated calomel electrode (SCE) and graphite rod were used as the reference electrode and the counter electrode, respectively. The reference electrode was also calibrated using the Pt RDE as working electrode in highly pure H_2_-saturated 1.0 M KOH solution and 0.5 M H_2_SO_4_ solution (Supplementary Fig. 13). All potentials were referenced to a reversible hydrogen electrode (RHE). Electrochemical impedance spectroscopy (EIS) measurements were performed at different potentials from 10^5^ to 0.1 Hz to obtain the solution resistance at a constant overpotential of 20 mV for HER and 200 mV for OER. The stability measurements were performed by cyclic voltammetry scanning 1000–3000 cycles (CV, sweep rate, 100 mV s^−1^).

### Electrochemical measurements for overall water splitting

The full electrolyzer configuration was assembled using two identical RuIrO_*x*_ electrodes and measured in a two-electrode cell with a carbon fiber paper (CFP) as the carrier. 5.0 mg of RuIrZnO_*x*_ pre-catalysts, 1.0 mg of pure carbon (Vulcan XC72), and 0.8 mg of polyvinylidene fluoride (PVDF) were dispersed in 1 mL water/ethanol (*v:v*, 1:4) solution by sonication to form a homogeneous ink. Then, 200 μL of the ink was daubed uniformly in 1 cm^2^ of area on a piece of 1 × 3 cm CFP (pre-catalyst loading: 1.0 mg cm^−2^). The polarization curves were measured at a scan rate of 5 mV s^−1^ in 0.5 M H_2_SO_4_, 1.0 M KOH and phosphate buffer solutions (PBS) with pH range from 3 to 12. The concentrations of the PBS are 1.0 M for pH = 3–9 and 0.8 M for pH = 12. The stability measurements were conducted by long-term chronoamperometry.

### Electrochemical surface area (ECSA) measurements

The underpotential deposition of copper (Cu-UPD) on Pt, Ru, and Ir has proven to be an ideal method for quantifying their corresponding electrochemical surface area (ECSA)^51–53^. In this approach, ECSA can be calculated based on the UPD copper stripping charge (Q_Cu_, Cu_UPD_ → Cu^2+^ + 2e^−^) with the following equation:

1 $${\mathrm{ECSA}} = \frac{{{\mathrm{Q}}_{{{\mathrm{Cu}}}}}}{{m \cdot 420\,{\mathrm{uC}} \cdot {\mathrm{cm}}^{ - 2}}}$$

### XAS measurements

The XAS (Ru K-edge and Ir L_3_-edge) spectra were collected at BL14W1 station in Shanghai Synchrotron Radiation Facility (SSRF, the storage rings were operated at 3.5 GeV with a maximum current of 250 mA). The data were collected at room temperature (Si (311) monochromator). The RuIrZnO_*x*_ pre-catalyst and the standard samples (Ru foil/RuO_2_/metallic Ir/IrO_2_) were tested in transmission mode, which were pelletized into disks of 13 mm diameter using graphite powder as a binder (ground thoroughly by mortar and pestle). The RuIrO_*x*_ after electrochemical activation were tested in fluorescence mode using the carbon fiber paper (CFP). 5.0 mg of RuIrZnO_*x*_ pre-catalysts, 1.0 mg of pure carbon (Vulcan XC72), and 0.8 mg of polyvinylidene fluoride (PVDF) were dispersed in 1 mL water/ethanol (*v:v*, 1:4) solution by sonication to form a homogeneous ink. Then, 200 μL of the ink was daubed uniformly in 1 cm^2^ of area on a piece of 1 × 3 cm CFP (pre-catalyst loading: 1.0 mg cm^−2^). The in situ generated RuIrO_*x*_ was obtained after running for several CV cycles within the potential range of specific electrochemical reactions.

### Operando XAS studies

Samples for operando XAS measurements were analyzed in a custom-made in situ cell at room temperature. The saturated calomel electrode (SCE) and graphite rod were used as the reference electrode and the counter electrode, respectively. The saturated calomel electrode was calibrated against an RHE before the operando measurements. The CFP loaded with RuIrO_*x*_ after the electrochemical activation was used as the working electrode. After electrochemical activation, XAS was collected at 0, −0.01 and −0.02 V (versus RHE), starting with the 0 V and stepping down to −0.02 V for HER condition. As for OER, XAS was collected at 1.0, 1.43, and 1.48 V (versus RHE), starting with the 1.0 V and stepping up to 1.48 V. At each potential, the catalyst was allowed to be stabilized 5 min before XAS measurement and the potential was kept constant throughout the XAS measurement.

### EXAFS analysis

The acquired EXAFS data were processed according to the standard procedures using the ATHENA module implemented in the IFEFFIT software packages. The EXAFS spectra were obtained by subtracting the post-edge background from the overall absorption and then normalizing with respect to the edge jump step. Then, *χ*(*k*) data in the *k*-space ranging from 2.2 to 12.3 Å^−1^ for Ru and 2.8 to 12.6 Å^−1^ for Ir were Fourier transformed to real (*R*) space using hanning windows (d*k* = 1.0 Å^−1^) to separate the EXAFS contributions from different coordination shells. The quantitative information can be obtained by the least-squares curve fitting in the *R* space with a Fourier-transform *k* space range 2.2–12.3 Å^−1^ for Ru and 2.8–12.6 Å^−1^ for Ir, using the module ARTEMIS of programs of IFEFFIT. The backscattering amplitude *F*(*k*) and phase shift Φ(*k*) were calculated using FEFF8.0 code.

### Computational details

Density functional theory (DFT) calculations were performed using the Vienna Ab-initio Simulation Package (VASP)^54,55^ with the projector augmented wave (PAW) method^56^ and a cutoff kinetic energy of 400 eV for plane-wave basis set. The generalized gradient approximation with PBE functional^57^ was used. An energy difference within 1.0 × 10^−5^ eV and force threshold of 0.02 eV/Å for the maximal component were set as the convergence criteria for solving for wavefunctions and geometry optimization, respectively. The reciprocal Brillouin zones were sampled by the Γ-centered Monkhorst-Pack scheme^58^ with resolutions of around 2π × 1/40 Å^−1^.

The 3 × 3 Ru (0001) and Ir (111) periodic slabs with six layers were utilized in the HER mechanism. The bottom two layers were fixed to the bulk positions, while the top four layers were allowed to relax. As the AC HAADF-STEM (Fig. 3c) shows that the Ir-incorporated RuIr system is of *fcc* phase, we built the RuIr model based on the *fcc* Ru surface by considering three configurations (Supplementary Fig. 36), with the ratio of Ru:Ir = 2:1, close to the experimental value of 2.01:1. The Zn-incorporated RuIrZn model was built by substituting one Ir/Ru atom with Zn atom at the surface or in the slab of the most stable Ru_2_Ir (111) surface.

The optimized lattice parameters of bulk rutile RuO_2_ were determined to be *a* = 4.505 Å and *c* = 3.119 Å, which are in good agreement with experimental results^59^. The models for Ir-incorporated RuO_*x*_ (Supplementary Fig. 41) was built by substituting Ru atoms with Ir atoms to reach a ratio of Ru:Ir = 1:1. In addition, we used the fully O-terminated phase in our calculations as previous work has shown that it is the most stable phase at high potentials^47^. All of the (110) facets were modeled by 2 × 1 supercells and four-layer slabs, with vacuum regions of 15 Å. The bottom two layers were fixed to the bulk positions, while the top two layers were allowed to relax. To simulate the influence of Zn doping on the catalytic performance, we substituted one Ru/Ir atom with Zn atom at the surface or in the slab of RuIrO_*x*_ (110) to mimic the RuIrZnO_*x*_ system.

The generally accepted acidic HER mechanism consists of two steps, with the Volmer step followed by the Heyrovsky step or Tafel step. So, HER can be described by the Volmer-Heyrovsky or Volmer-Tafel mechanism.

2 $$\left( {{\mathrm{Volmer}}\,{\mathrm{step}}} \right) ^{\ast} + {\mathrm{H}}_3{\mathrm{O}}^ + + {\mathrm{e}}^ - \to {}^{\ast} {\mathrm{H}} + {\mathrm{H}}_2{\mathrm{O}}$$

3 $$\left( {{\mathrm{Heyrovsky}}\,{\mathrm{step}}} \right) ^{\ast} {\mathrm{H}} + {\mathrm{H}}_3{\mathrm{O}}^ + + {\mathrm{e}}^ - \to {}^{\ast} + {\mathrm{H}}_2 + {\mathrm{H}}_2{\mathrm{O}}$$

4 $$\left( {{\mathrm{Tafel}}\,{\mathrm{step}}} \right)\,2 ^{\ast} {\mathrm{H}} \to 2 ^{\ast} + {\mathrm{H}}_2$$

The alkaline HER can be also described by the Volmer-Heyrovsky or Volmer-Tafel mechanism with the following steps.

5 $$\left( {{\mathrm{Volmer}}\,{\mathrm{step}}} \right)^{\ast} + {\mathrm{H}}_2{\mathrm{O}} + {\mathrm{e}}^ - \to {^{\ast} \mathrm{H}} + {\mathrm{OH}}^ - $$

6 $$\left( {{\mathrm{Heyrovsky}}\,{\mathrm{step}}} \right) ^{\ast} {\mathrm{H}} + {\mathrm{H}}_2{\mathrm{O}} + {\mathrm{e}}^ - \to {}^{\ast} + {\mathrm{H}}_2 + {\mathrm{OH}}^ - $$

and the Tafel step (Eq. (4)).

We consider the H adsorption as the key descriptor for HER as suggested previously^46^. We studied the reaction mechanisms to understand the performance of different ruthenium oxide (RuO_*x*_) catalysts for driving the OER. Previous work has proved that the oxygen coupling (*O + *O) reaction is less favorable than the formation of *OOH intermediate^47^. Thus, we assumed a four-step water hydrogen atom abstraction mechanism under acidic OER condition involving the following steps, where the symbol “*” represents the active site (five-coordinated Ir, Ru, or Zn atom).

7 $$ ^{\ast} + {\mathrm{H}}_2{\mathrm{O}} \to {^{\ast}\mathrm{OH}} + {\mathrm{H}}^ + + {\mathrm{e}}^ - $$

8 $$ ^{\ast} {\mathrm{OH}} \to {^{\ast}\mathrm{O}} + {\mathrm{H}}^ + + {\mathrm{e}}^ - $$

9 $$ ^{\ast} {\mathrm{O}} + {\mathrm{H}}_2{\mathrm{O}} \to {^{\ast}\mathrm{OOH}} + {\mathrm{H}}^ + + {\mathrm{e}}^ - $$

10 $$ ^{\ast} {\mathrm{OOH}} \to {}^{\ast} + {\mathrm{O}}_2 + {\mathrm{H}}^ + + {\mathrm{e}}^ - $$

The OER mechanism under alkaline condition can be described by the following four steps.

11 $$ ^{\ast} + {\mathrm{OH}}^ - \to {}^{\ast} {\mathrm{OH}} + {\mathrm{e}}^ - $$

12 $$ {^{\ast}\mathrm{OH}} + {\mathrm{OH}}^ - \to {^{\ast}\mathrm{O}} + {\mathrm{H}}_2{\mathrm{O}} + {\mathrm{e}}^ - $$

13 $$ {^{\ast}\mathrm{O}} + {\mathrm{OH}}^ - \to {^{\ast}\mathrm{OOH}} + {\mathrm{e}}^ - $$

14 $$ {^{\ast}\mathrm{OOH}} + {\mathrm{OH}}^ - \to ^{\ast}+ {\mathrm{O}}_2 + {\mathrm{H}}_2{\mathrm{O}} + {\mathrm{e}}^ - $$

## Supplementary information

Supplementary Information

Supplementary Information - Movie

## Data Availability

The data that support the findings of this study are available from the corresponding authors upon a reasonable request.
