# Supplementary material for: Three-dimensional open nano-netcage electrocatalysts for efficient pH-universal overall water splitting
Source: Nat Commun. 2019 Oct 25;10:4875. doi: 10.1038/s41467-019-12885-0 (PMC6814841; doi:10.1038/s41467-019-12885-0)
Supplement: Supplementary file 1 — Supplementary Information [file 41467_2019_12885_MOESM1_ESM.pdf]

Supplementary Information

Three-dimensional open nano-netcage electrocatalysts for efficient pH-universal overall water splitting

Zhuang et al.

Supplementary Figures

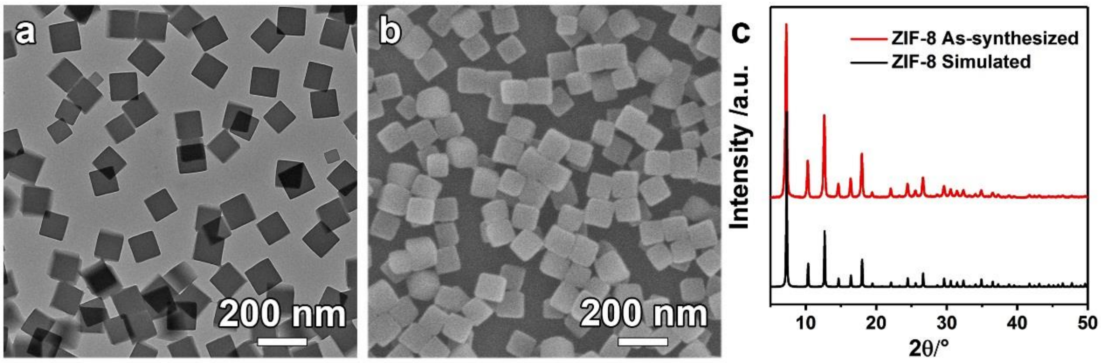

Supplementary Figure 1. Characterizations of ZIF-8 nanocubes. a, TEM image. **b**, SEM image. **c**, XRD patterns of ZIF-8 nanocubes.

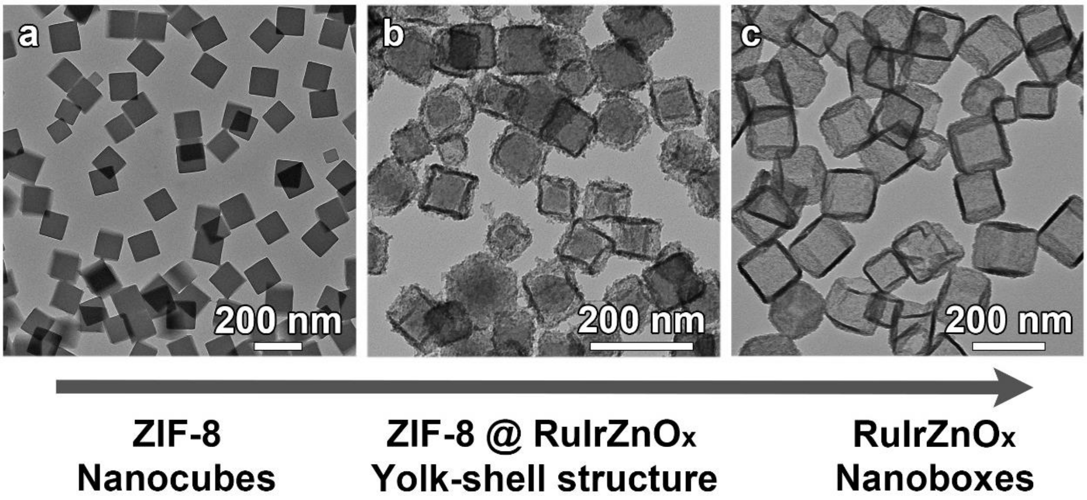

Supplementary Figure 2. TEM images of the samples obtained at three representative stages during the evolution process from ZIF-8 nanocubes to hollow nanoboxes. **a**, Initial ZIF-8 nanocubes. **b**, Intermediates obtained after 5 mins of solvothermal reaction. **c**, Products obtained after 2 hr of solvothermal reaction.

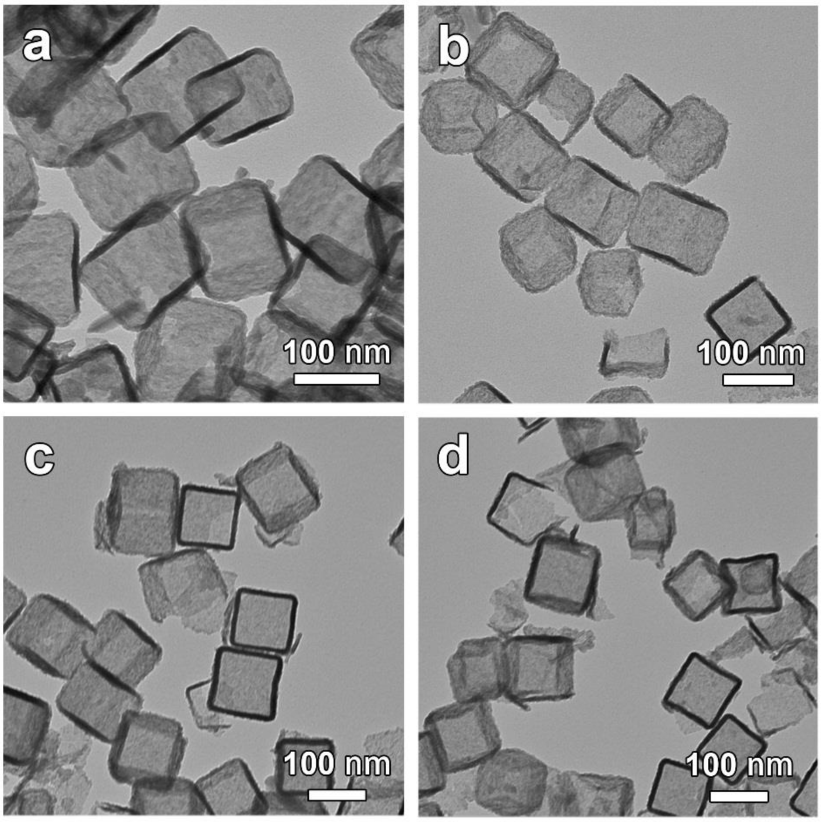

Supplementary Figure 3. TEM images of samples with different Ru:Ir ratios. a, $RuZnO_x$. b, $RuIrZnO_x$-25. c, $RuIrZnO_x$-50. d, $RuIrZnO_x$-72.5. 25, 50 and 72.5 indicates the introduction amount of Ir precursor (μmol). $RuIrZnO_x$-72.5 was simply denoted as $RuIrZnO_x$ in the manuscript.

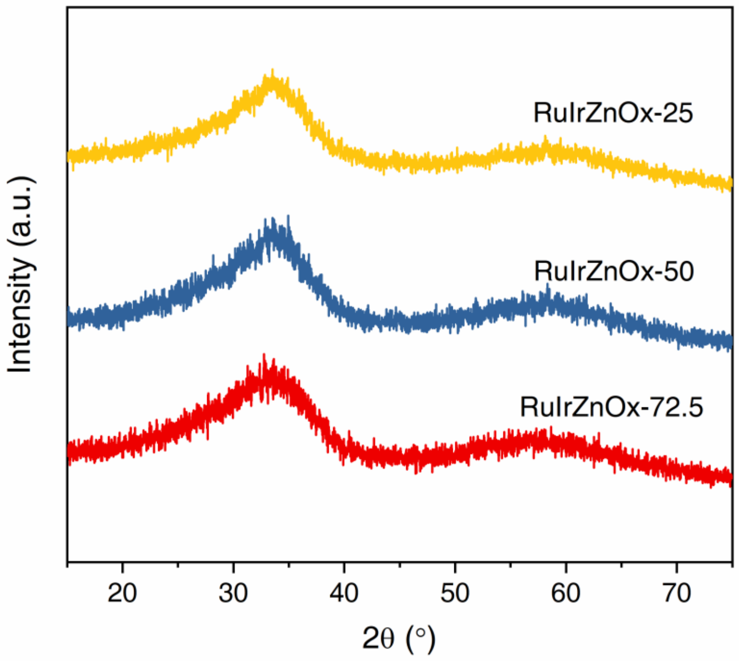

Supplementary Figure 4. XRD patterns of samples with different Ru:Ir ratios.

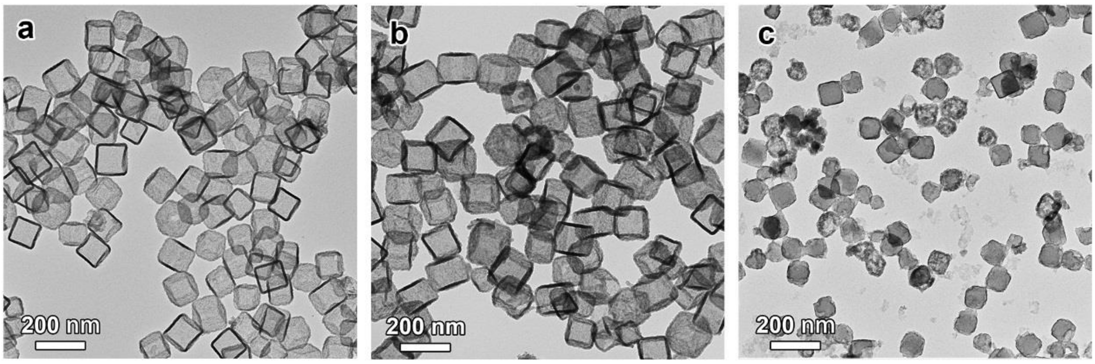

Supplementary Figure 5. Solvothermal reaction products of ZIF-8 with (a) Ru only, (b) Ru + Ir, and (c) Ir only.

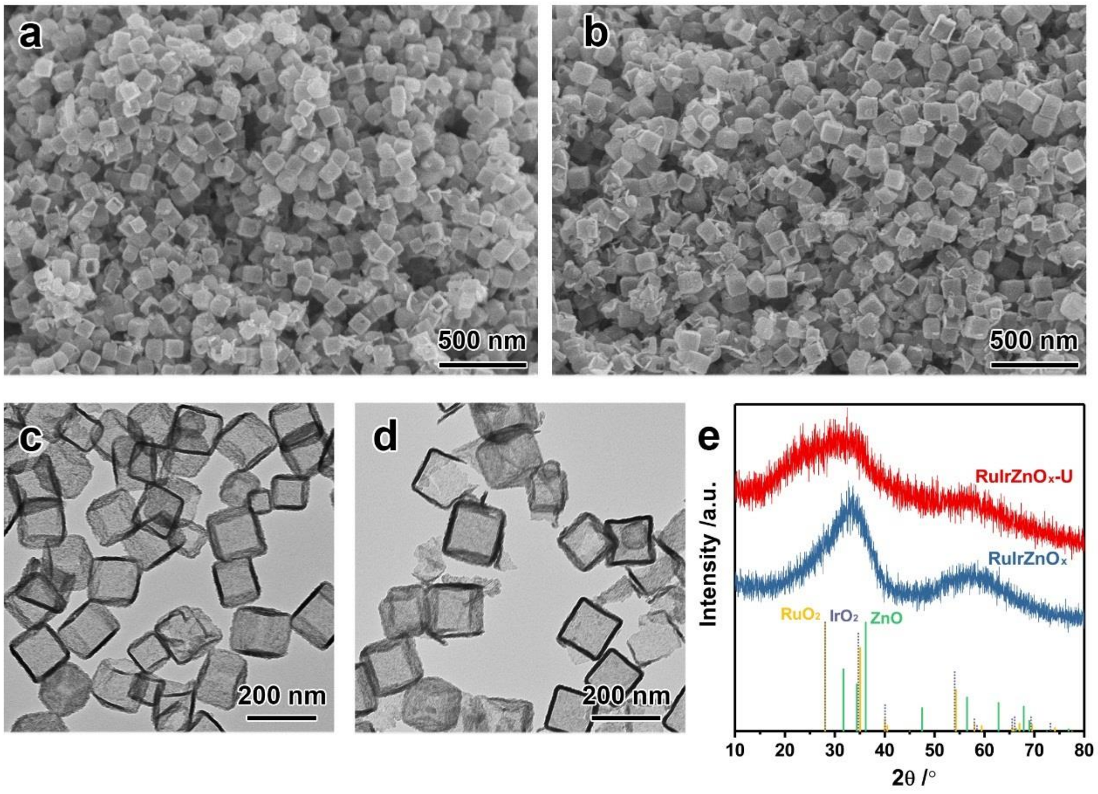

Supplementary Figure 6. Characterization of RuIrZnO$_x$-U and RuIrZnO$_x$. a-b, SEM images of RuIrZnO$_x$-U (**a**) and RuIrZnO$_x$ (**b**). **c-d,** TEM images of RuIrZnO$_x$-U (**c**) and RuIrZnO$_x$ (**d**). **e,** XRD patterns.

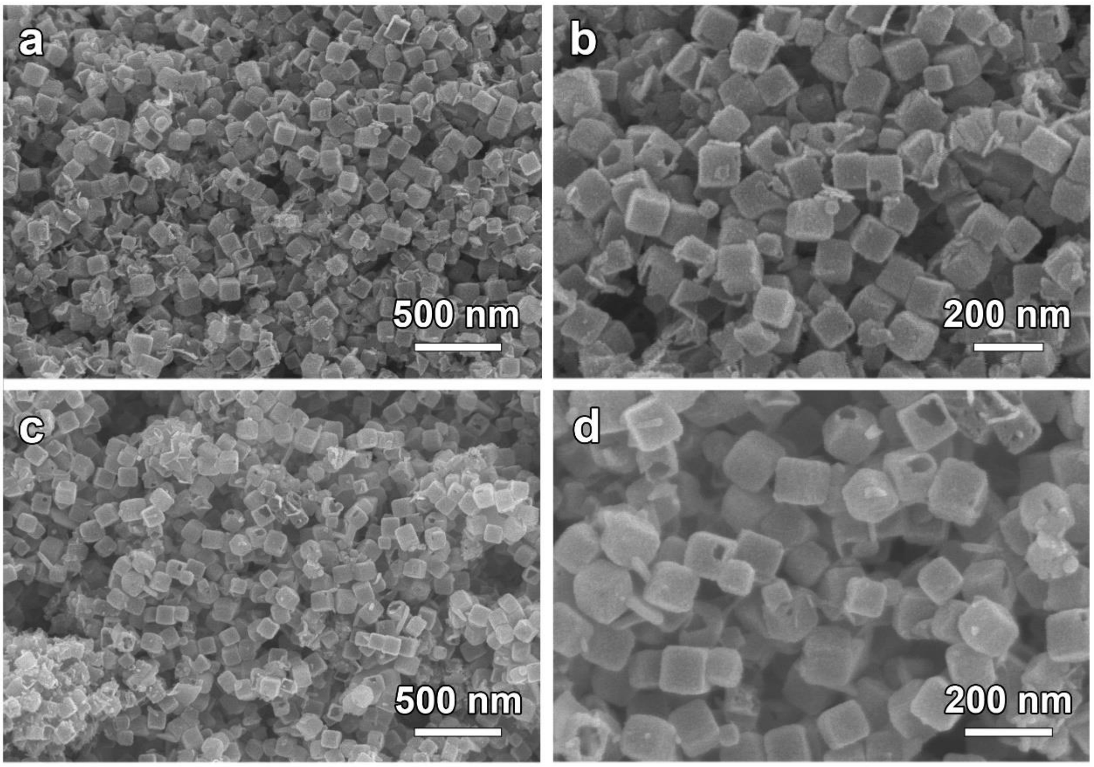

Supplementary Figure 7. SEM images. (**a**) Low, (**b**) high magnification SEM images of $RuIrZnO_x$ and (**c**) low, (**d**) high magnification SEM images of $RuZnO_x$.

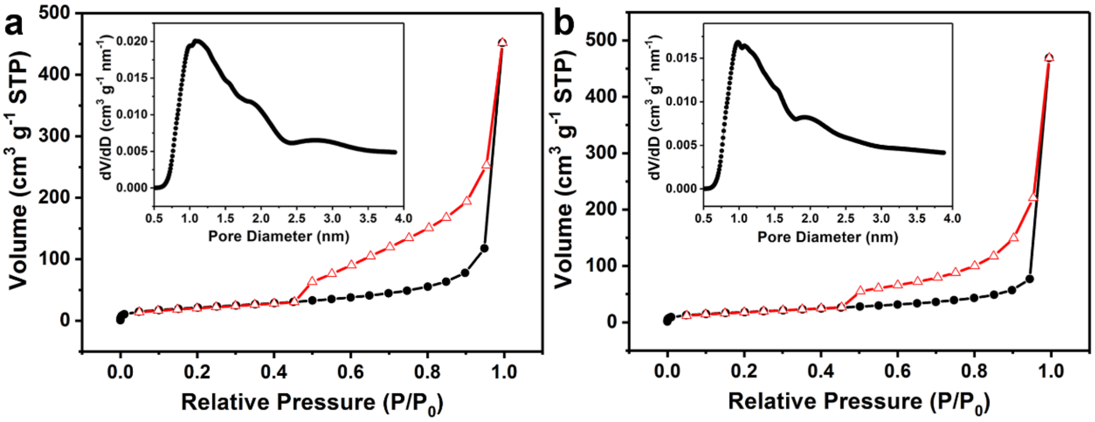

Supplementary Figure 8. Nitrogen adsorption-desorption isotherms. a, $RuIrZnO_x$. **b**, $RuZnO_x$. The insets are the corresponding micropore size distribution curves.

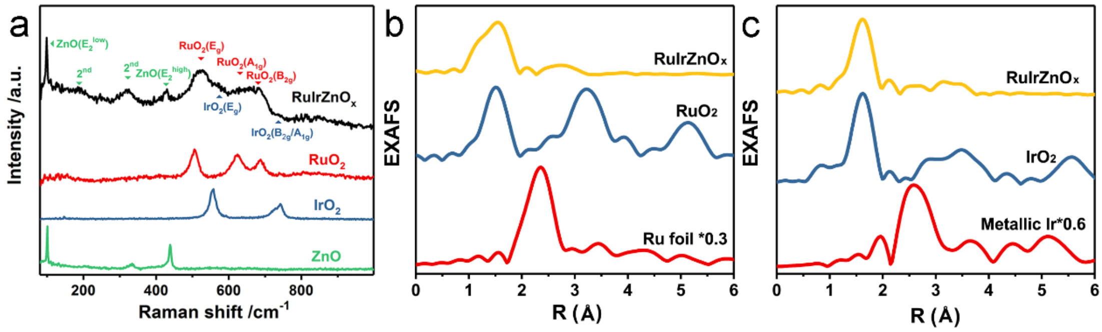

Supplementary Figure 9. X-ray absorption spectroscopic studies of the RuIrZnO*x*. **a,** Raman spectrum profile of RuIrZnO*x* nanobox. **b,** Fourier-transform EXAFS spectrum of RuIrZnO*x* nanobox in comparison with that of RuO_2 and Ru foil at the Ru K-edge. **c,** Fourier-transform EXAFS spectrum of RuIrZnO*x* nanobox in comparison with IrO_2 and Ir foil at the Ir L_3-edge.

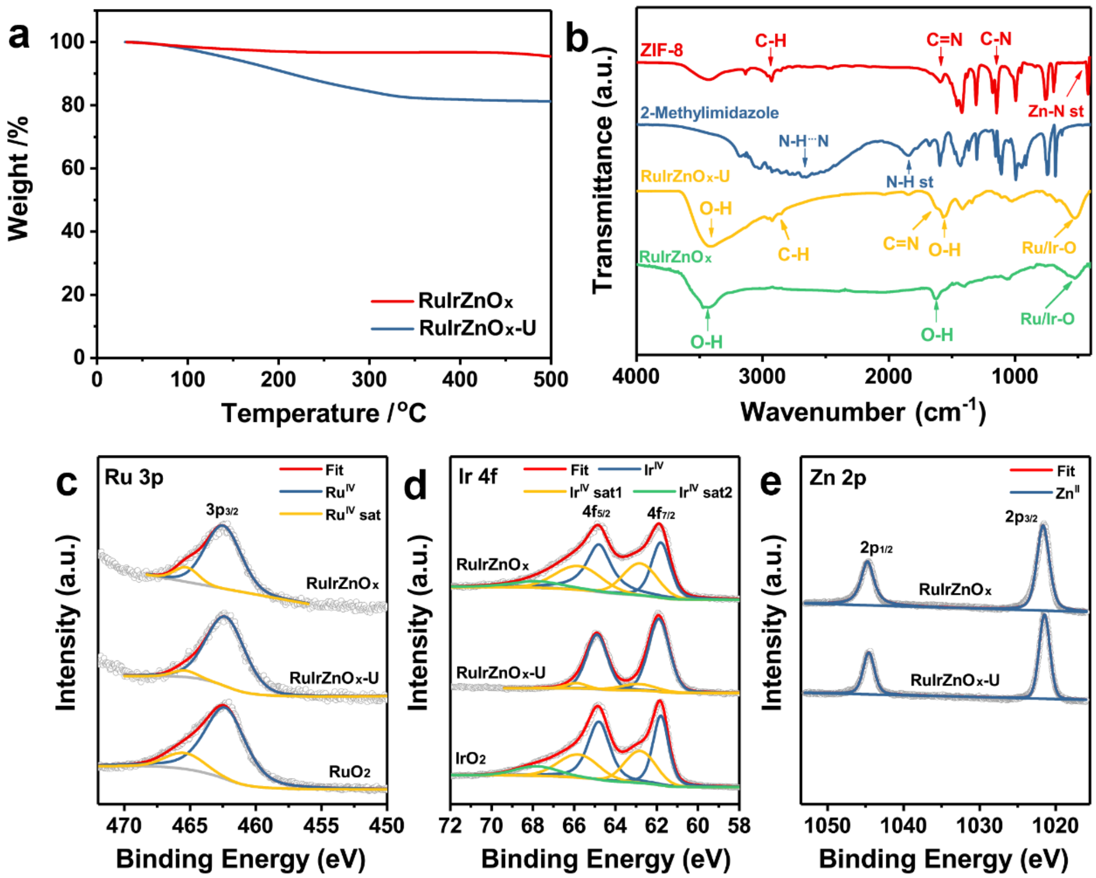

Supplementary Figure 10. Supplementary characterizations of of RuIrZnO$_x$-U and RuIrZnO$_x$. a, TGA profile. **b,** FT-IR spectra. **c-e,** XPS spectra.

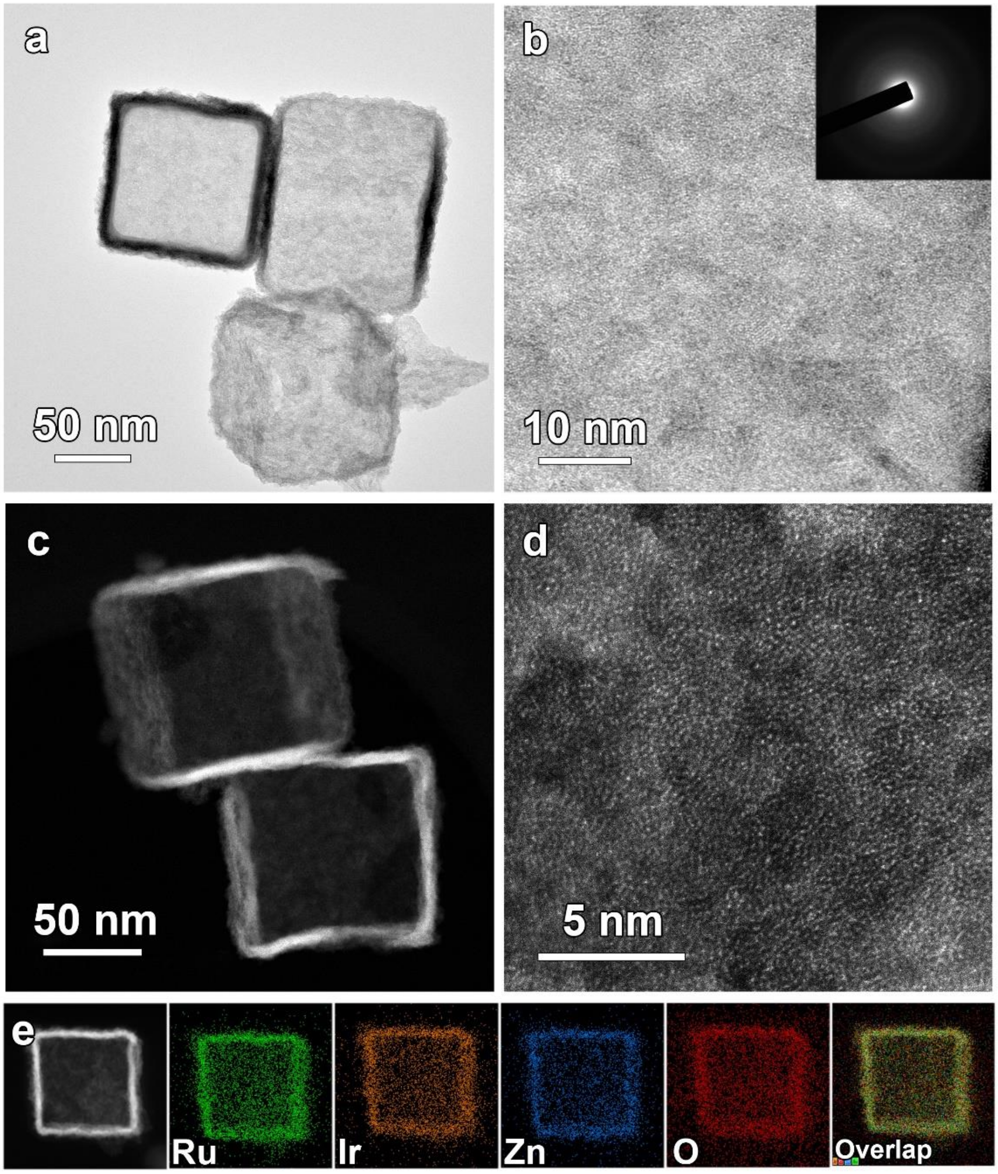

Supplementary Figure 11. Characterization of RuIrZnO$_x$ nanoboxes. a, HRTEM and **b**, magnified images. **c**, AC HAADF-STEM and **d**, magnified images. **e**, EDX elemental mapping. Inset of b shows the SAED pattern.

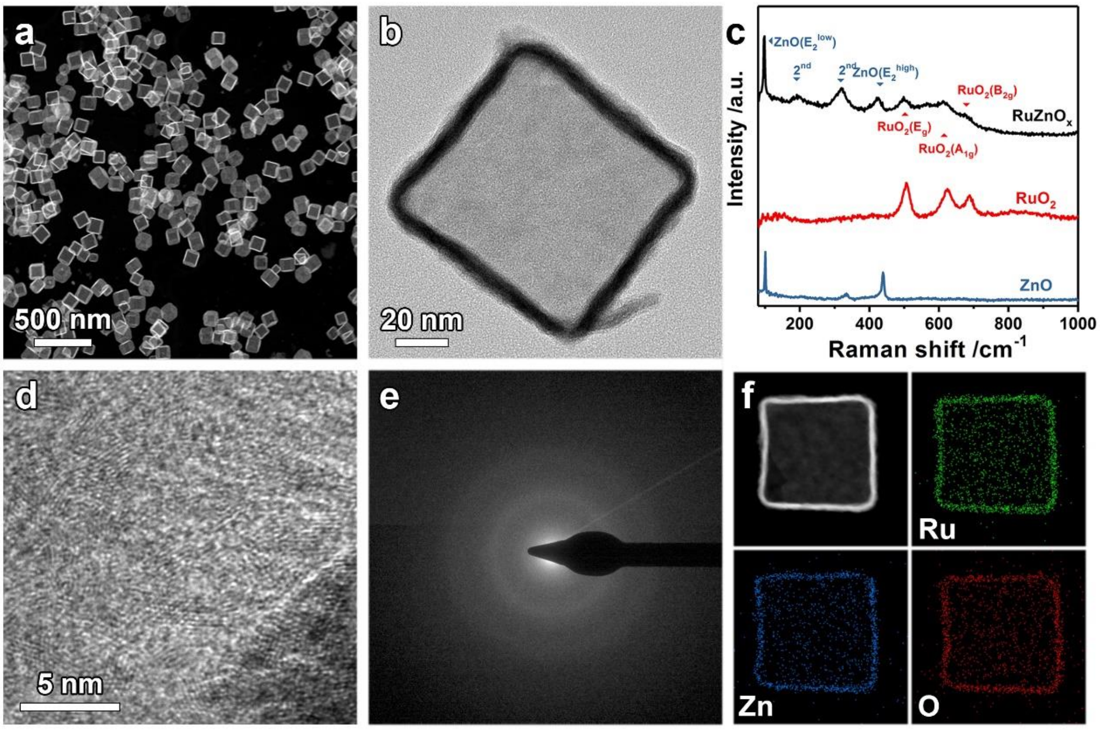

Supplementary Figure 12. Characterization of RuZnO$_x$ nanoboxes. a, Dark-filed HRTEM. **b**, Bright-filed HRTEM. **c**, Raman spectrum. **d**, Magnified HRTEM images. **e**, SAED. **f**, EDX elemental mapping.

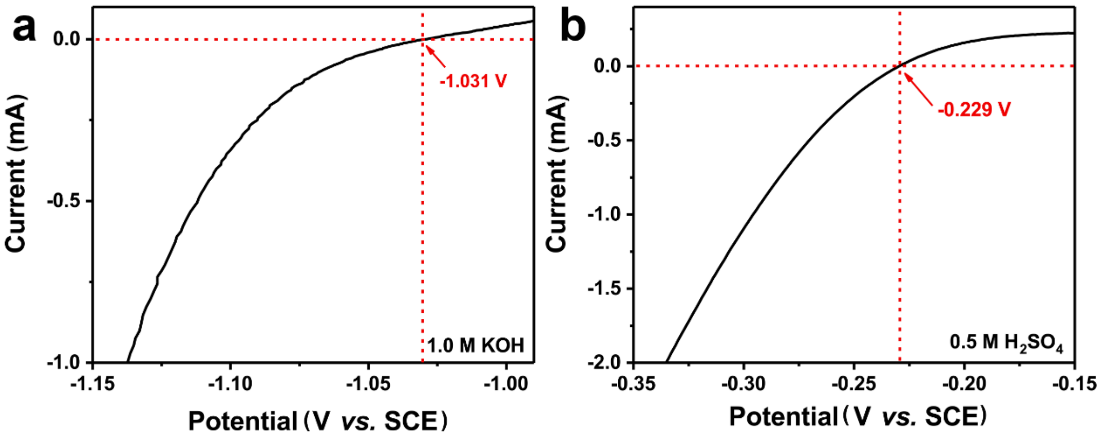

Supplementary Figure 13. Calibration of the saturated calomel electrode (SCE) with the respect to RHE. a, **b**, Current-potential curves of the Pt plate (1 cm^2) in highly pure H_2-saturated 1.0 M KOH solution and 0.5 M H_2SO_4 solution. Scan rate: 1 $mV \cdot s^{-1}$. All the measured polarization curve potentials in this work were converted to reverse hydrogen electrode (RHE) by following equations: $E_{RHE} = E_{SCE} + 1.031V$ (1.0 M KOH solution); $E_{RHE} = E_{SCE} + 0.229V$ (0.5 M H_2SO_4 solution).

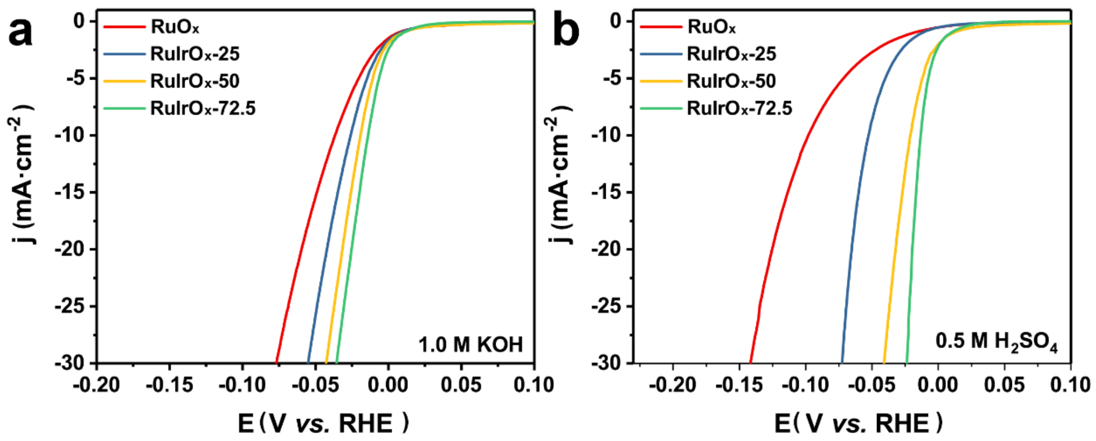

Supplementary Figure 14. HER performance of samples with different Ru:Ir ratios. a, **b**, Polarization curves of samples in 1.0 M KOH solution and 0.5 M H_2SO_4 solution. Scan rate: 1 mV·s^{-1}. The HER performance is enhanced as the Ir ratio increases. Therefore, we selected the RuIrZnO$_x$-72.5 with the saturated Ir ratio as the optimized catalyst for further investigation, which was simply denoted as RuIrZnO$_x$ in the manuscript.

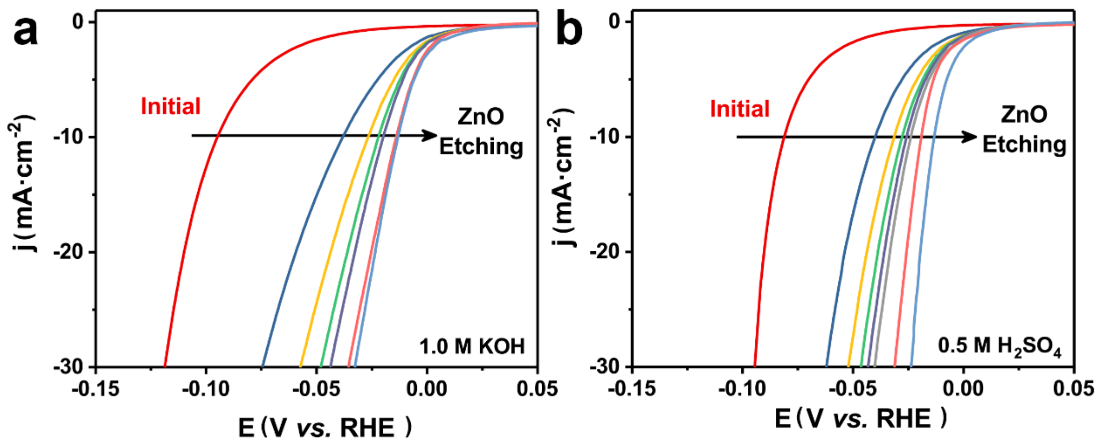

Supplementary Figure 15. HER activity trends as the etching of ZnO progressed. **a**, **b**, Polarization curves of $RuIrZnO_x$ *h*-nanoboxes in 1.0 M KOH solution and 0.5 M H_2SO_4 solution. Scan rate: 5 mV·s^{-1}.

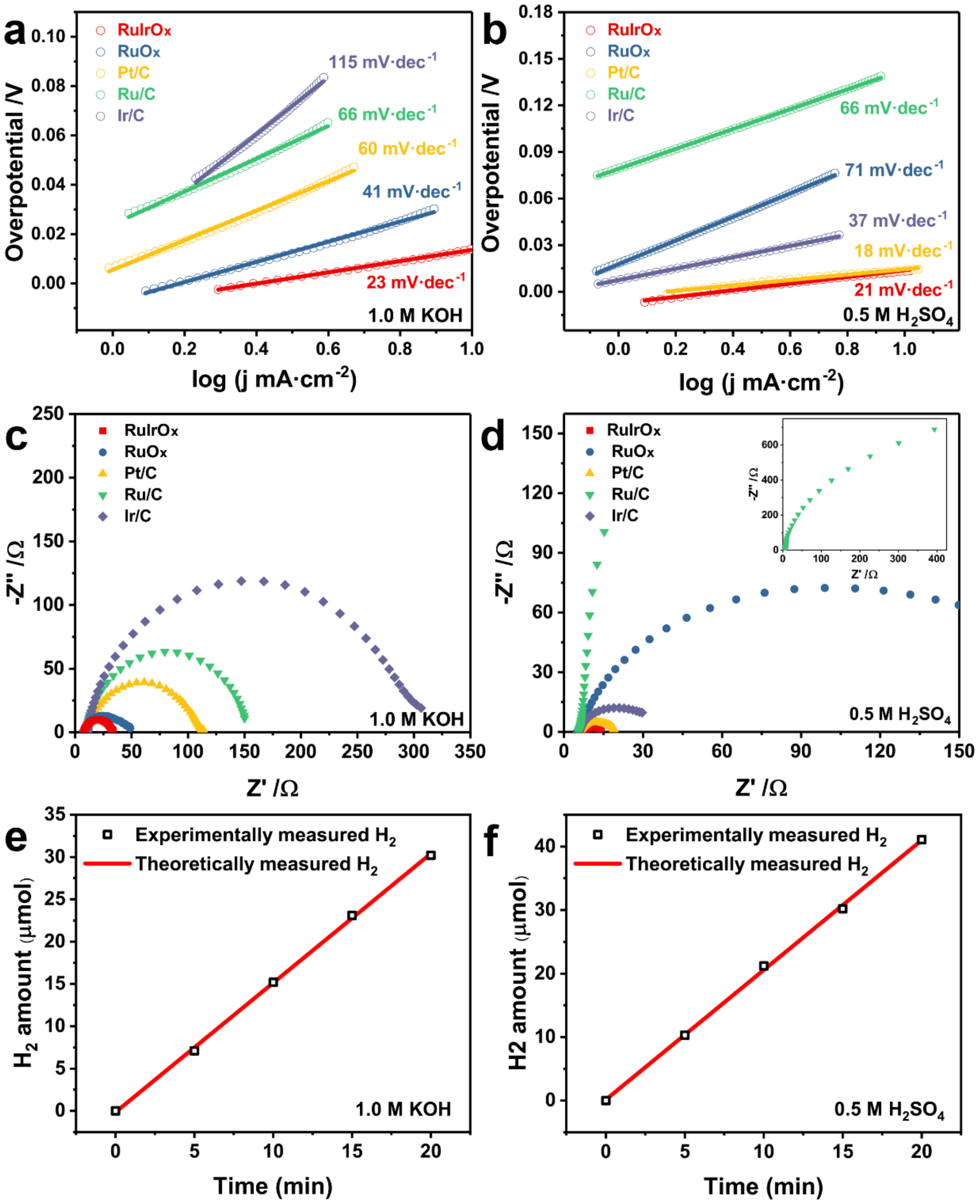

Supplementary Figure 16. HER electrochemical performance of typical samples. **a**, **b**, Tafel plots of electrocatalysts in 1.0 M KOH and 0.5 M H_2SO_4. **c**, **d**, Nyquist plots of the catalysts under the overpotential of 20 mV for HER in 1.0 M KOH solution and 0.5 M H_2SO_4 solution. **e**, **f**, Faradaic efficiencies.

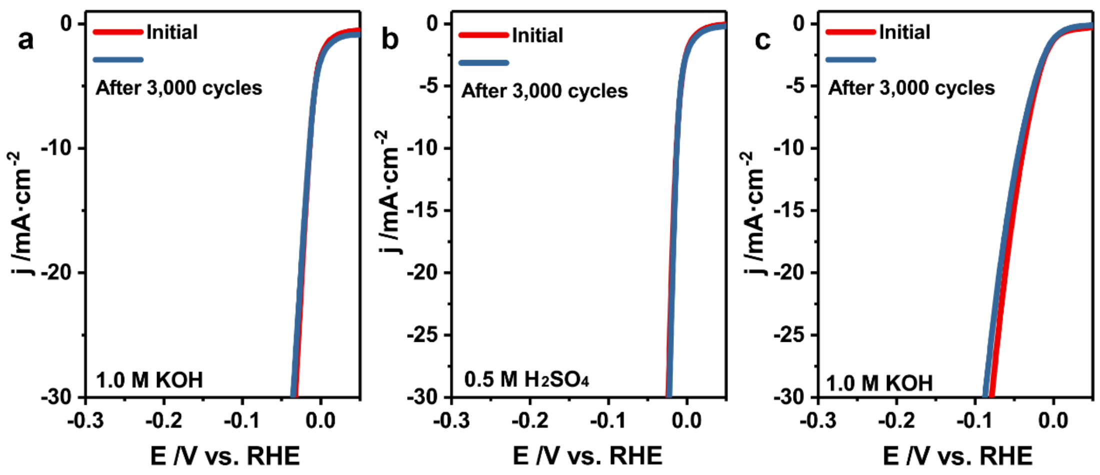

Supplementary Figure 17. HER Durability tests. a-b, The polarization curves of $RuIrO_x$ before and after 3,000 CV cycles in 1.0 M KOH solution and 0.5 M H_2SO_4 solution. **c**, The polarization curves of RuO_x before and after 3,000 CV cycles in 1.0 M KOH solution. Scan rate: 5 $mV{\cdot}s^{-1}$.

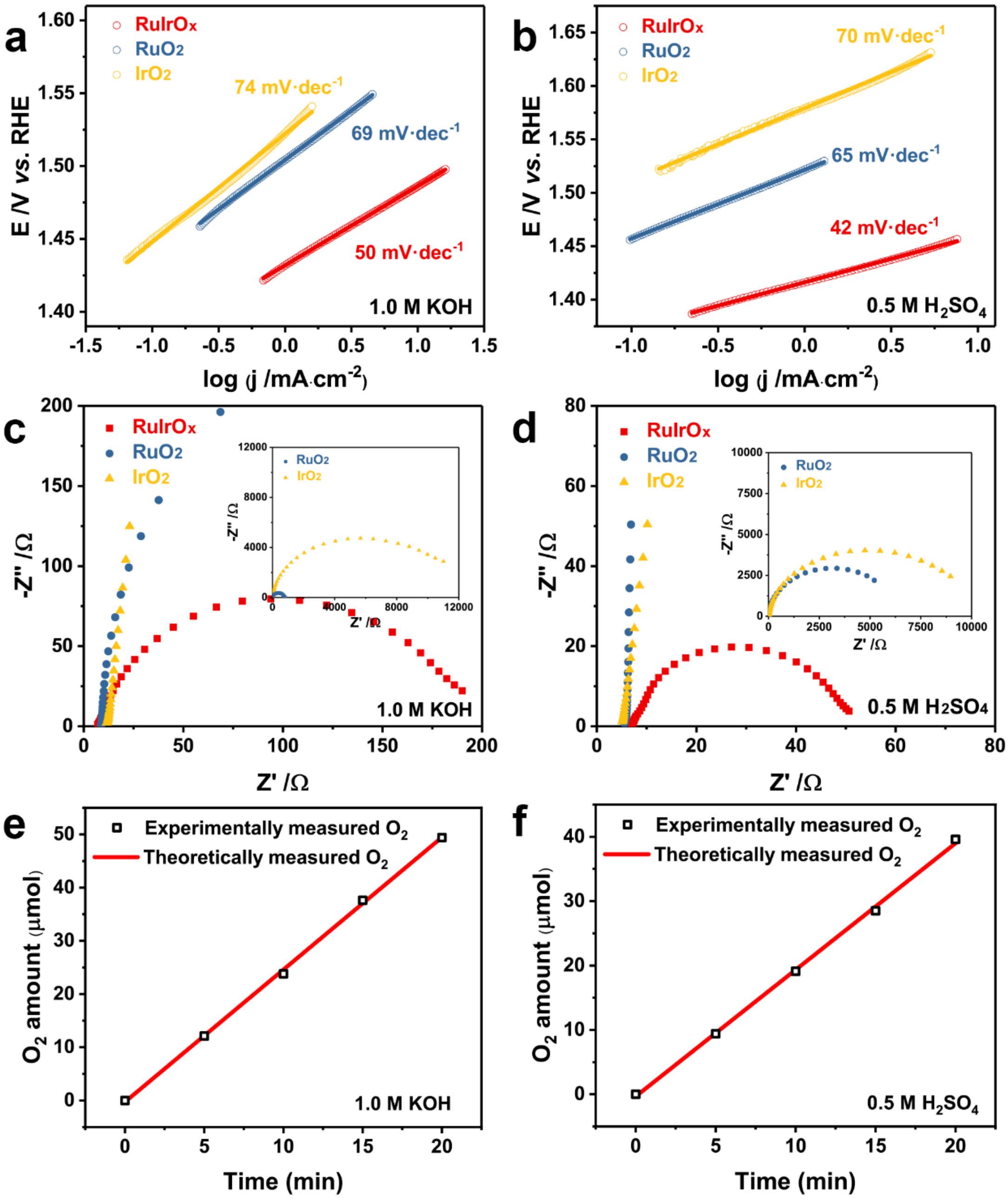

Supplementary Figure 18. OER electrochemical performance of typical samples. **a**, **b**, Tafel plots of electrocatalysts in 1.0 M KOH and 0.5 M H_2SO_4. **c**, **d**, Nyquist plots of the catalysts under the overpotential of 200 mV for OER in 1.0 M KOH solution and 0.5 M H_2SO_4 solution. **e**, **f**, Faradaic efficiencies.

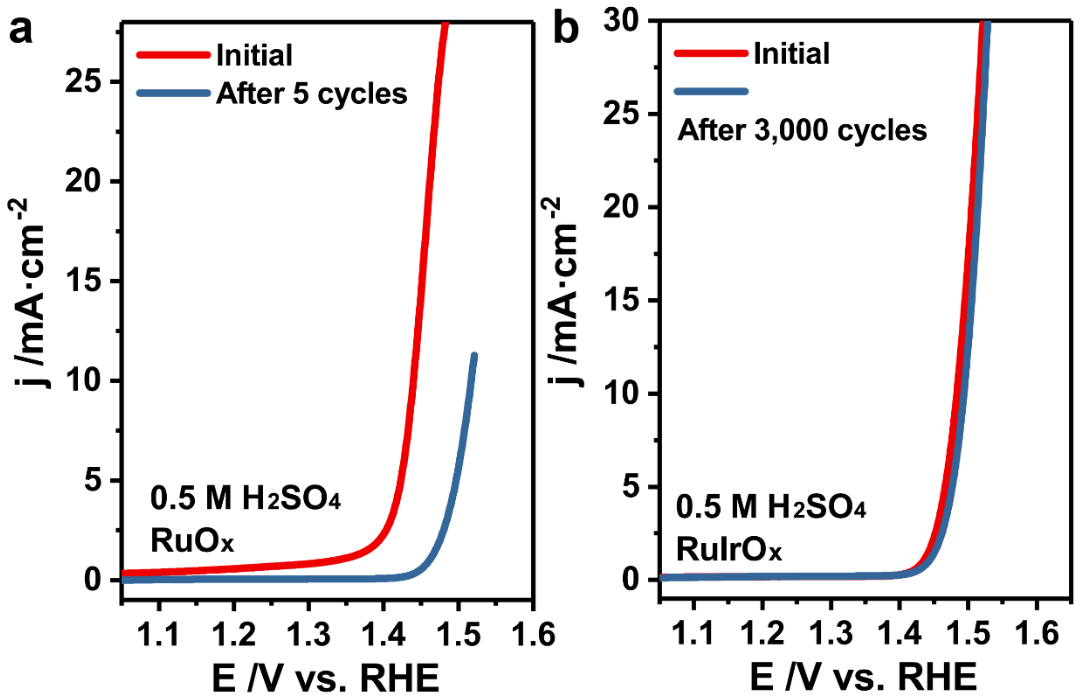

Supplementary Figure 19. OER Durability tests of RuIrO*$_x$* and RuO*$_x$* in 0.5 M H_2SO_4 solution. **a**, The polarization curves before and after 5 CV cycles of RuO$_x$. **b**, The polarization curves before and after 3,000 CV cycles of RuIrO$_x$ nano-netcage. Scan rate: 5 $mV{\cdot}s^{-1}$.

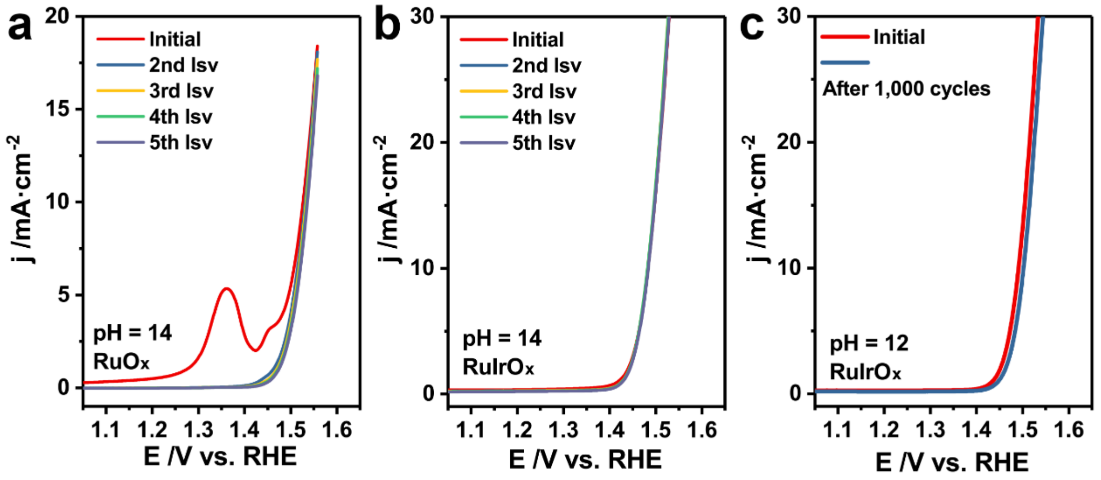

Supplementary Figure 20. OER Durability tests of RuIrO$_x$ and RuO$_x$ in alkaline solution. **a**, The polarization curves of RuO$_x$ in 1.0 M KOH solution (pH=14). **b**, The polarization curves of RuIrO$_x$ in 1.0 M KOH solution (pH=14). **c**, The polarization curves before and after 1,000 CV cycles of RuIrO$_x$ in PBS solution (pH=12). Scan rate: 5 $mV{\cdot}s^{-1}$.

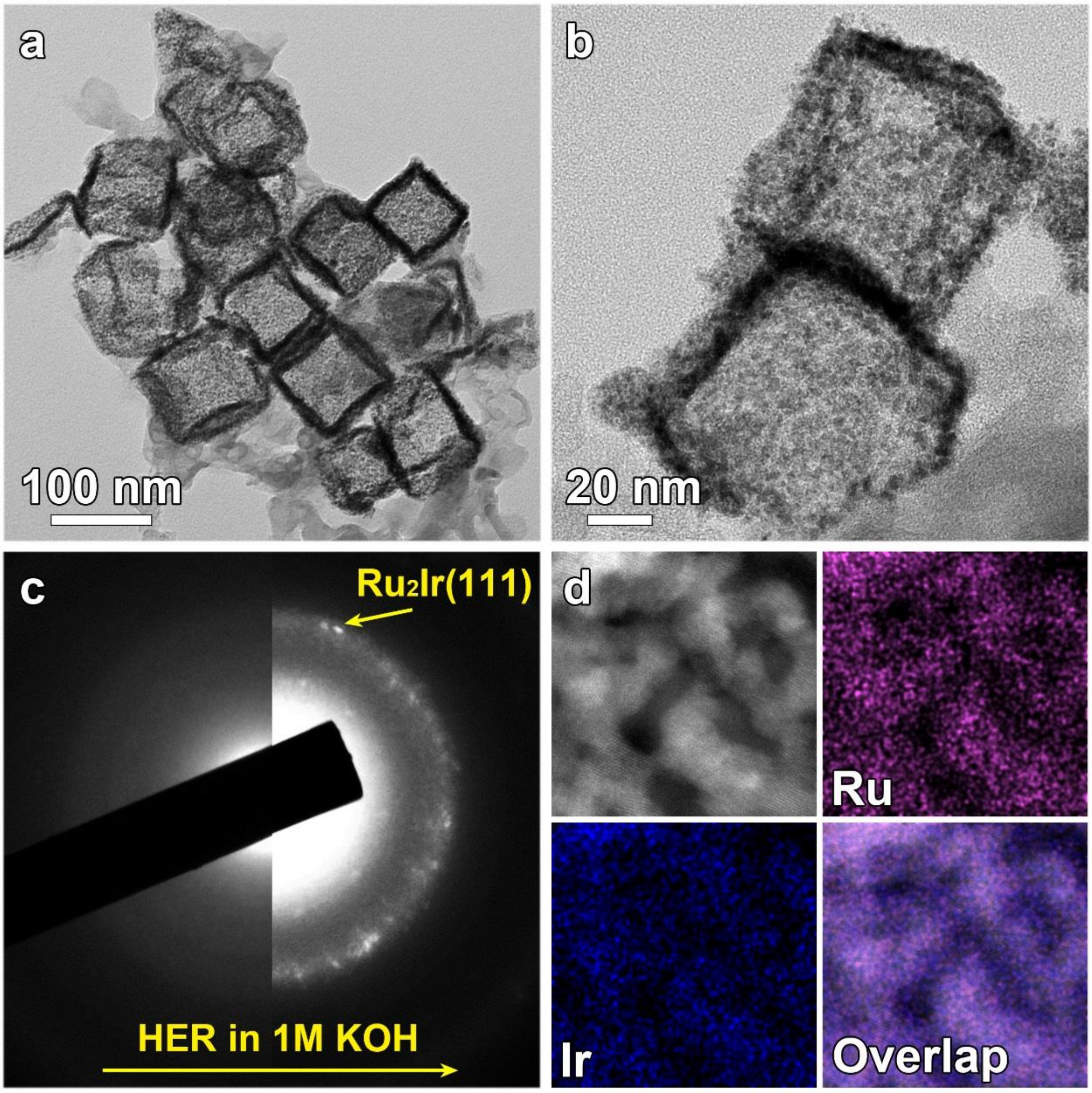

Supplementary Figure 21. Characterization of RuIrO$_x$-H-Alkaline. a, TEM. **b,** HRTEM. **c,** SAED. **d,** EDX elemental mapping.

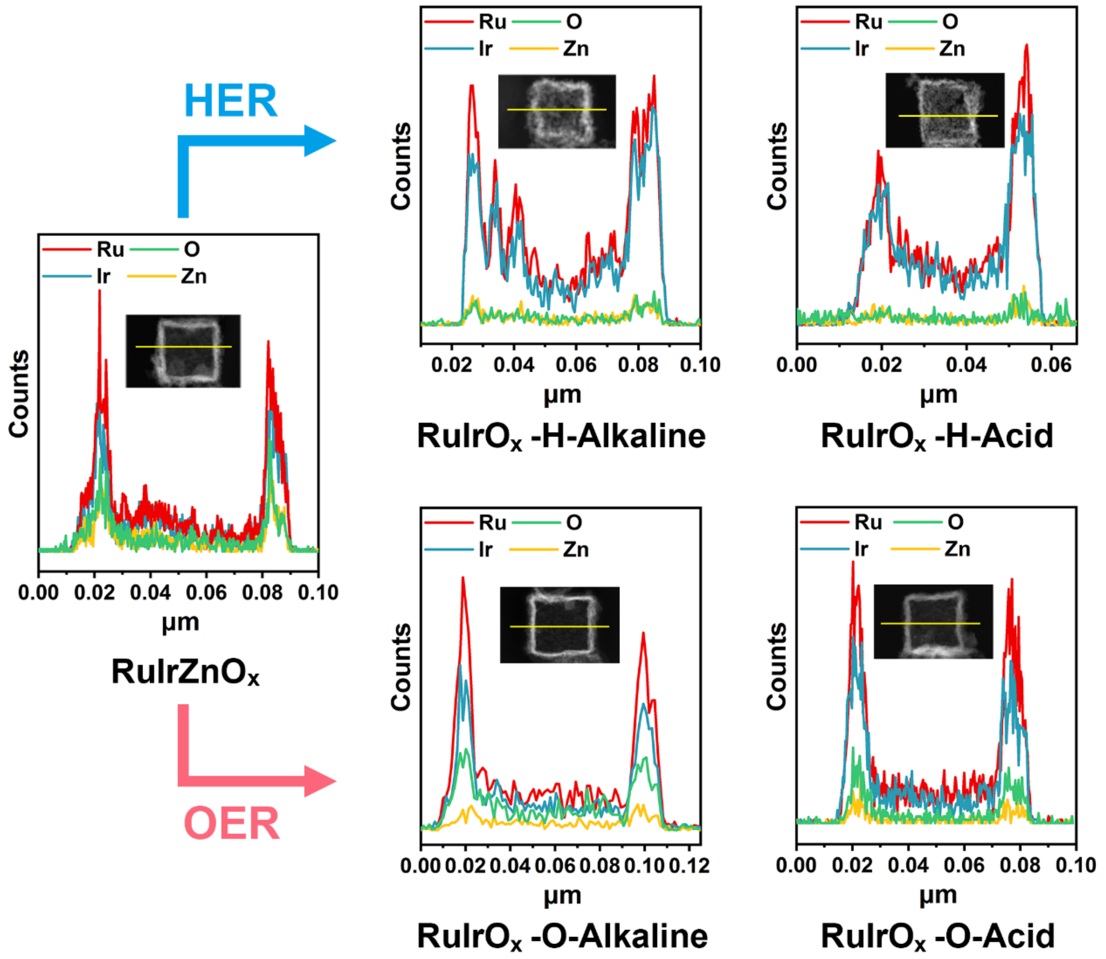

Supplementary Figure 22. Line-scan profiles of $RuIrZnO_x$ and $RuIrO_x$ after electrochemical activation.

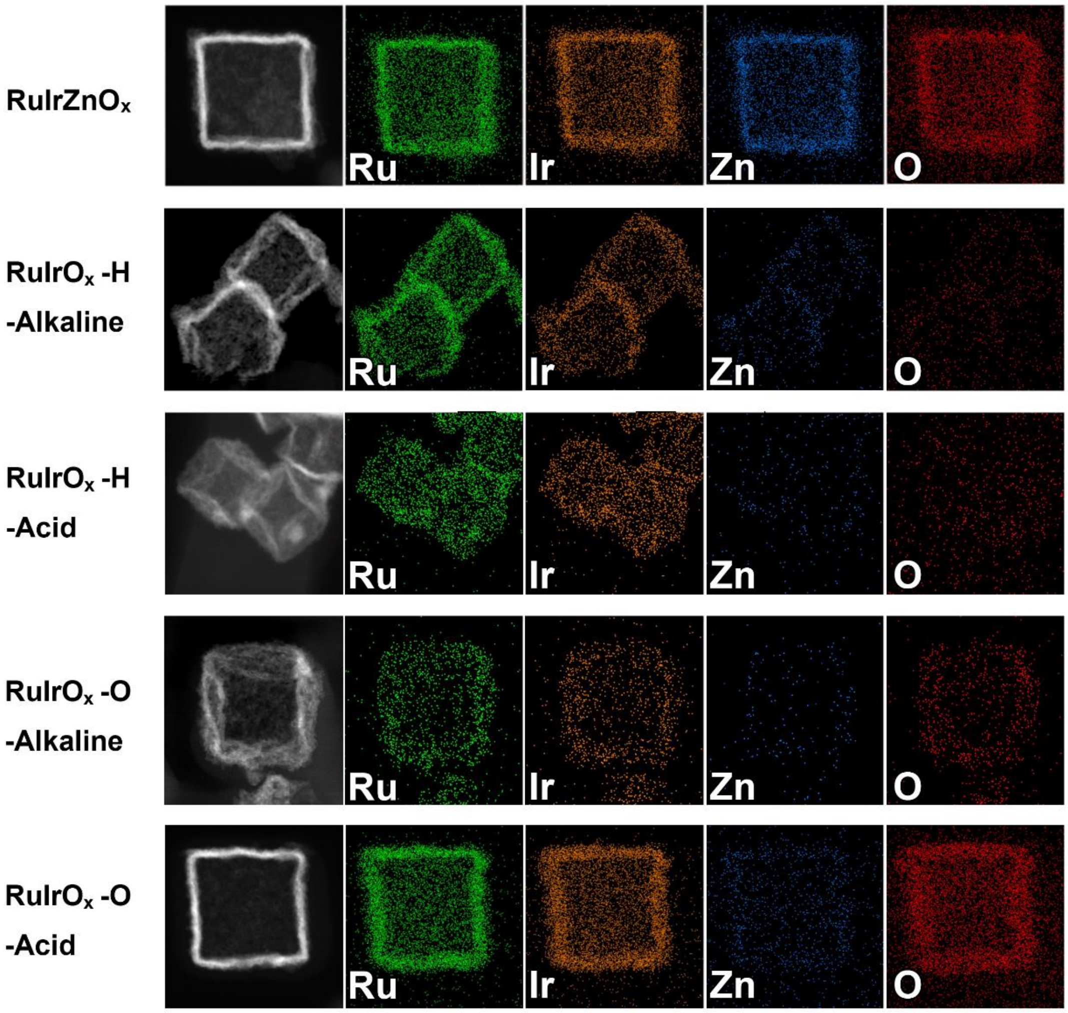

Supplementary Figure 23. EDX elemental mapping of RuIrZnO$_x$ and RuIrO$_x$ after electrochemical activation.

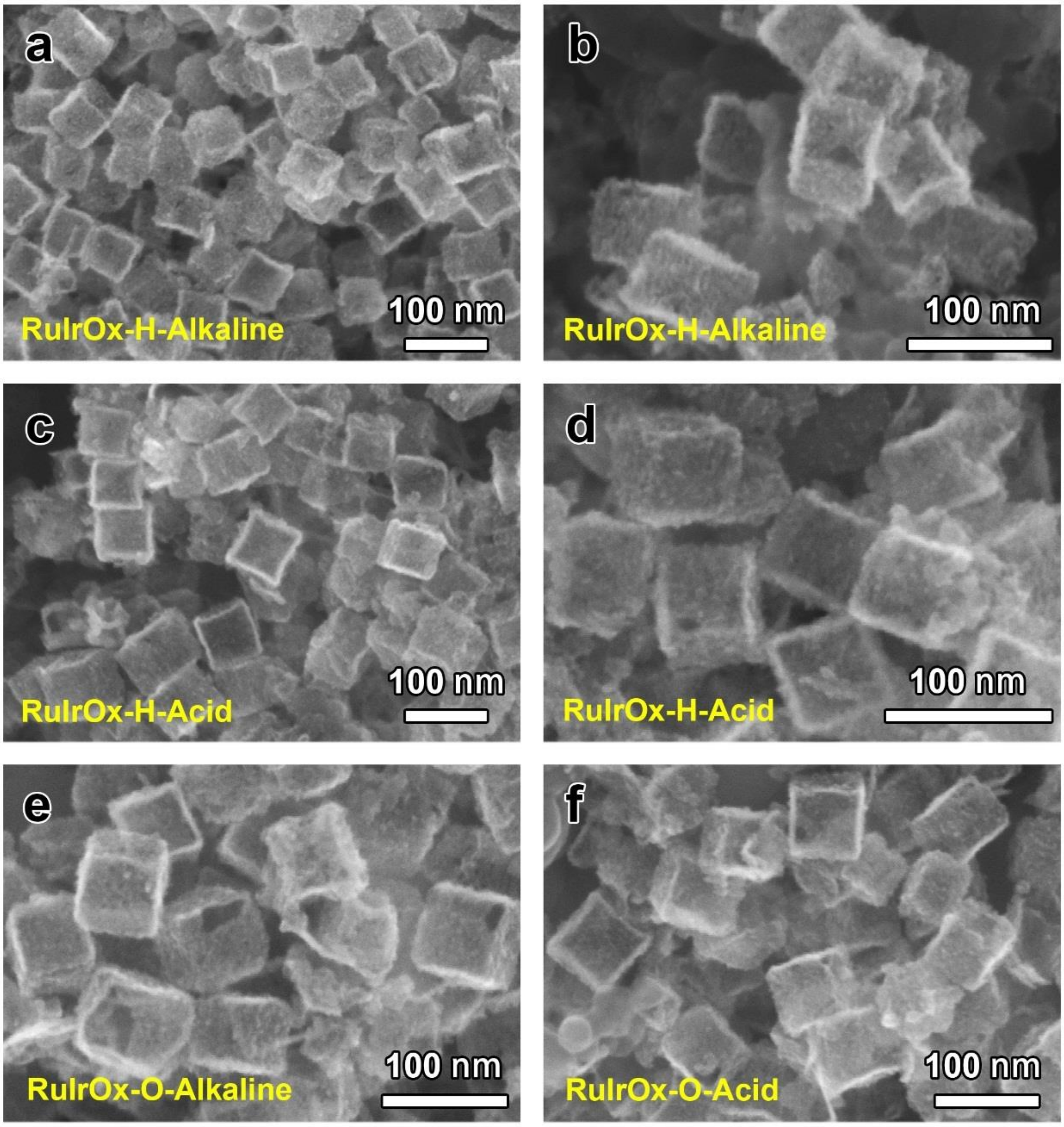

Supplementary Figure 24. SEM images of electrochemically *in-situ* formed RuIrO*x*.

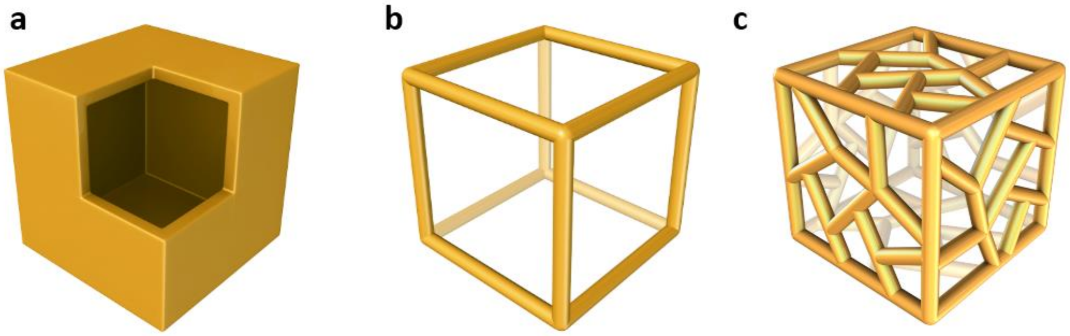

Supplementary Figure 25. Comparison of nanobox, nanoframe and nano-netcage.

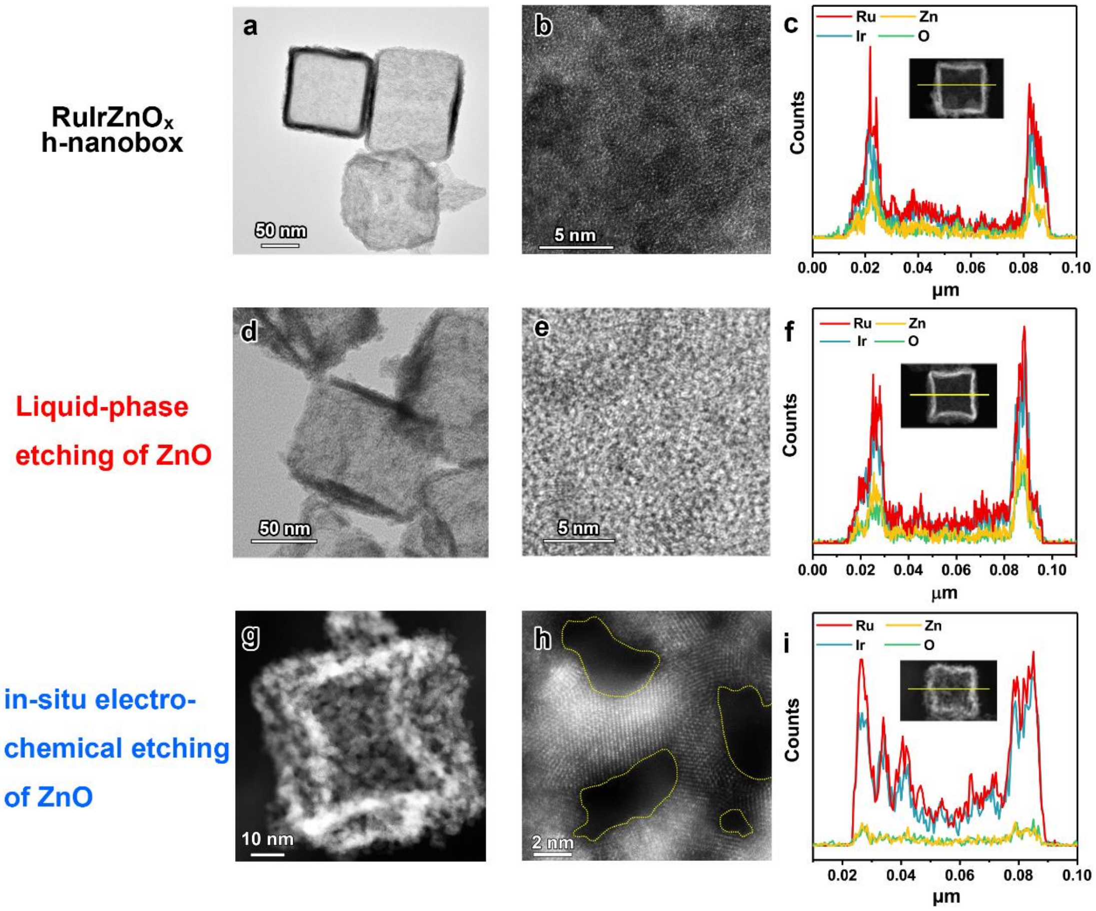

Supplementary Figure 26. Comparison of the products obtained from liquid-phase etching method and *in-situ* electrochemical etching method. a-c, HRTEM image (a), AC HAADF-STEM image **(b)** and EDX spectroscopy line-scan profiles **(c)** of $RuIrZnO_x$ h-nanobox. **d-f**, HRTEM images **(d-e)** and EDX spectroscopy line-scan profiles **(f)** of samples after liquid-phase etching of ZnO. **g-i**, AC HAADF-STEM images **(g-h)** and EDX spectroscopy line-scan profiles **(i)** of $RuIrO_x$ nano-netcage after *in-situ* electrochemical etching of ZnO. In a typical process, the $RuIrZnO_x$ nanoboxes (20 mg) were added into 10 mL 1 M KOH solution and stirred for 30 min. The nanostructures obtained from KOH etching have smooth surfaces, rather than porous ones. EDX line-scan revealed the existence of residual ZnO.

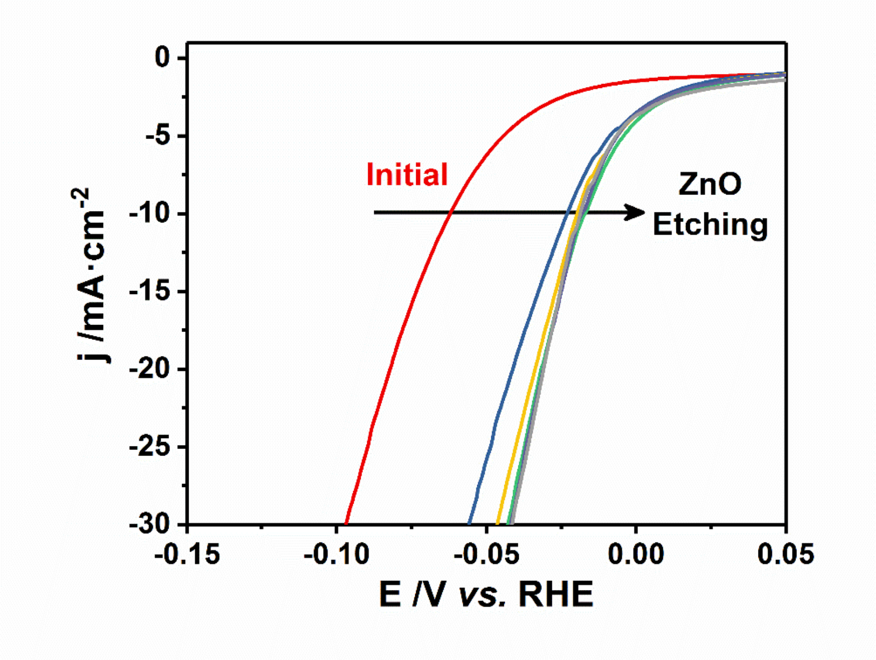

Supplementary Figure 27. HER of RuIrZnO$_x$ obtained after liquid-phase etching.

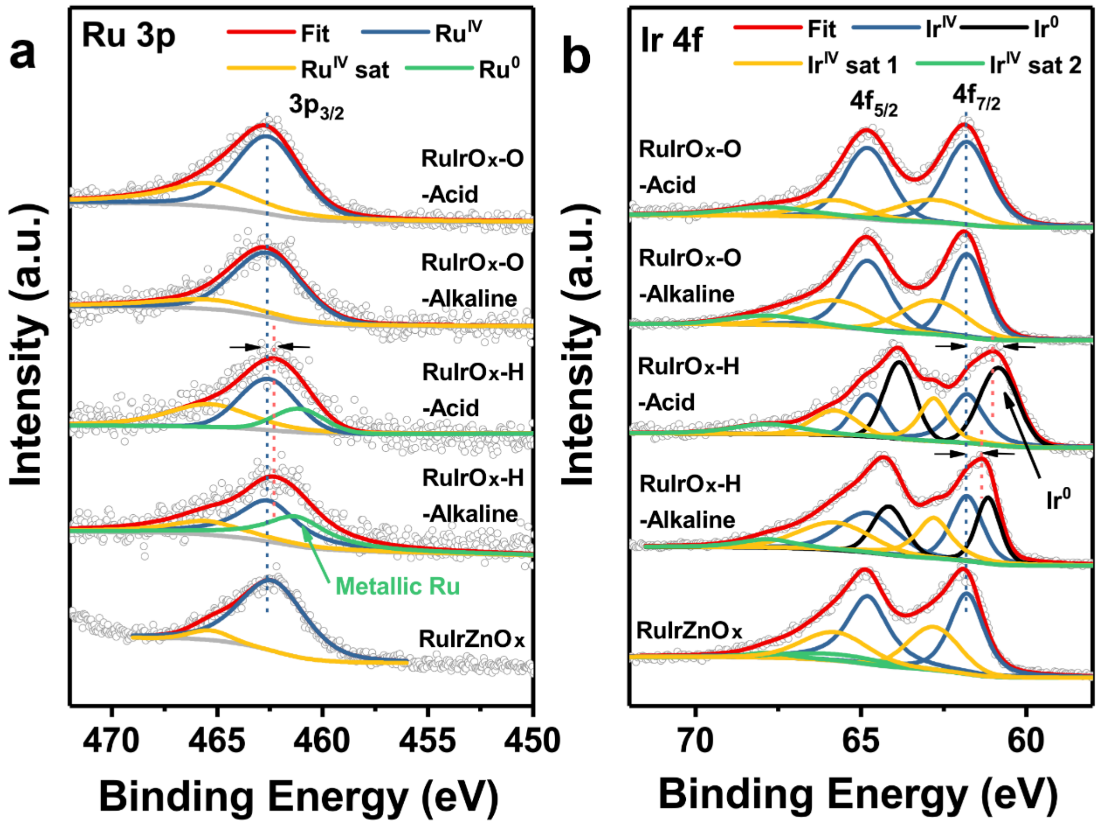

Supplementary Figure 28. XPS of electrochemical in-situ generated RuIrO$_x$.

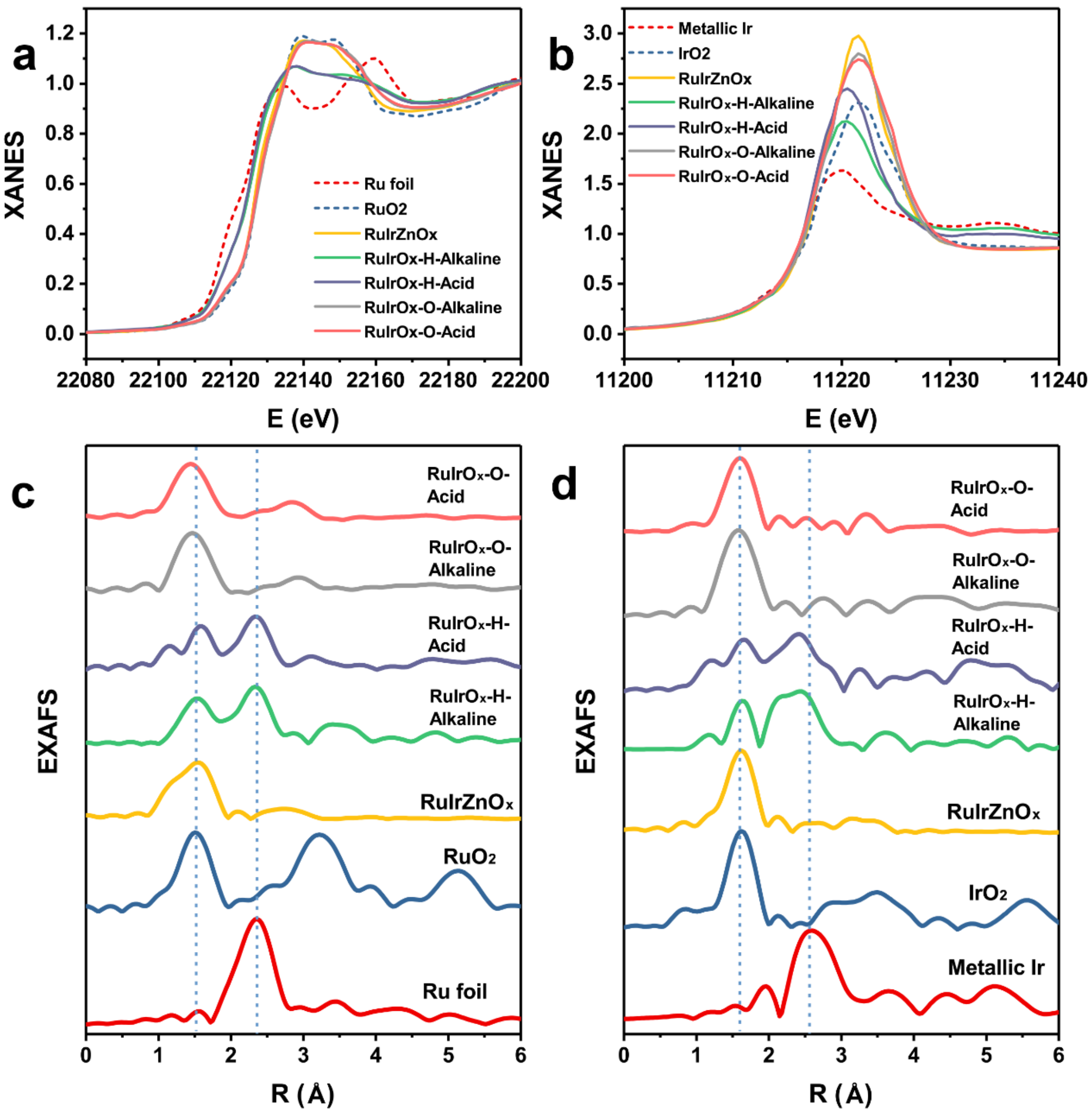

Supplementary Figure 29. XAS studies of electrochemically in-situ formed RuIrO$_x$.

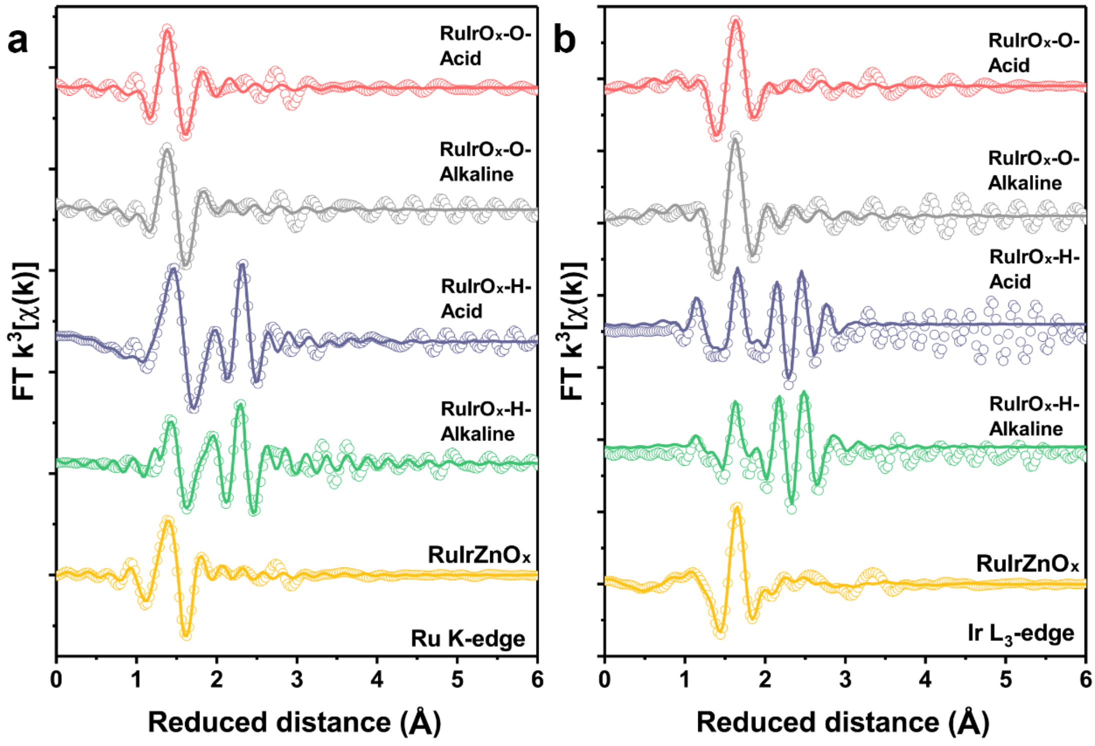

Supplementary Figure 30. k^3-weighted EXAFS oscillations collected at Ru K-edge (a) and Ir-L_3 edge (b) of different samples. The experimental (open symbols) and fits (solid lines) are shown.

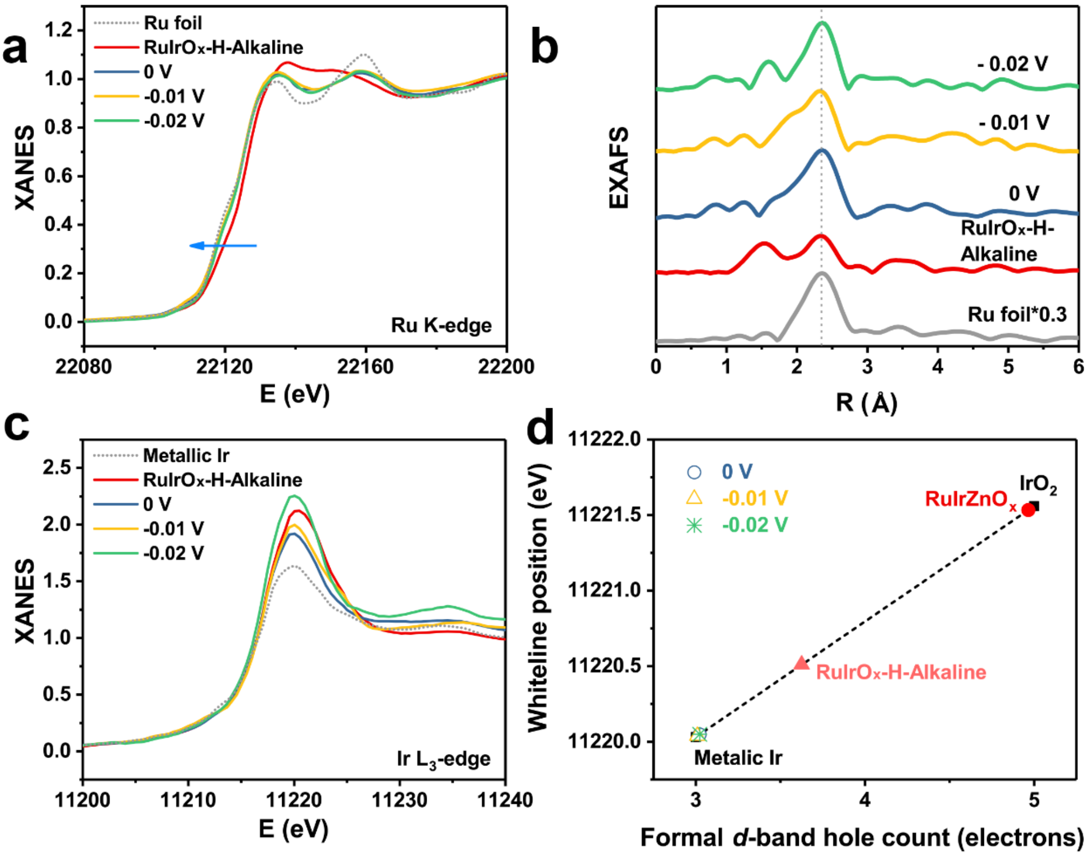

Supplementary Figure 31. operando XAS of $RuIrO_x$ under HER condition in 1 M KOH.

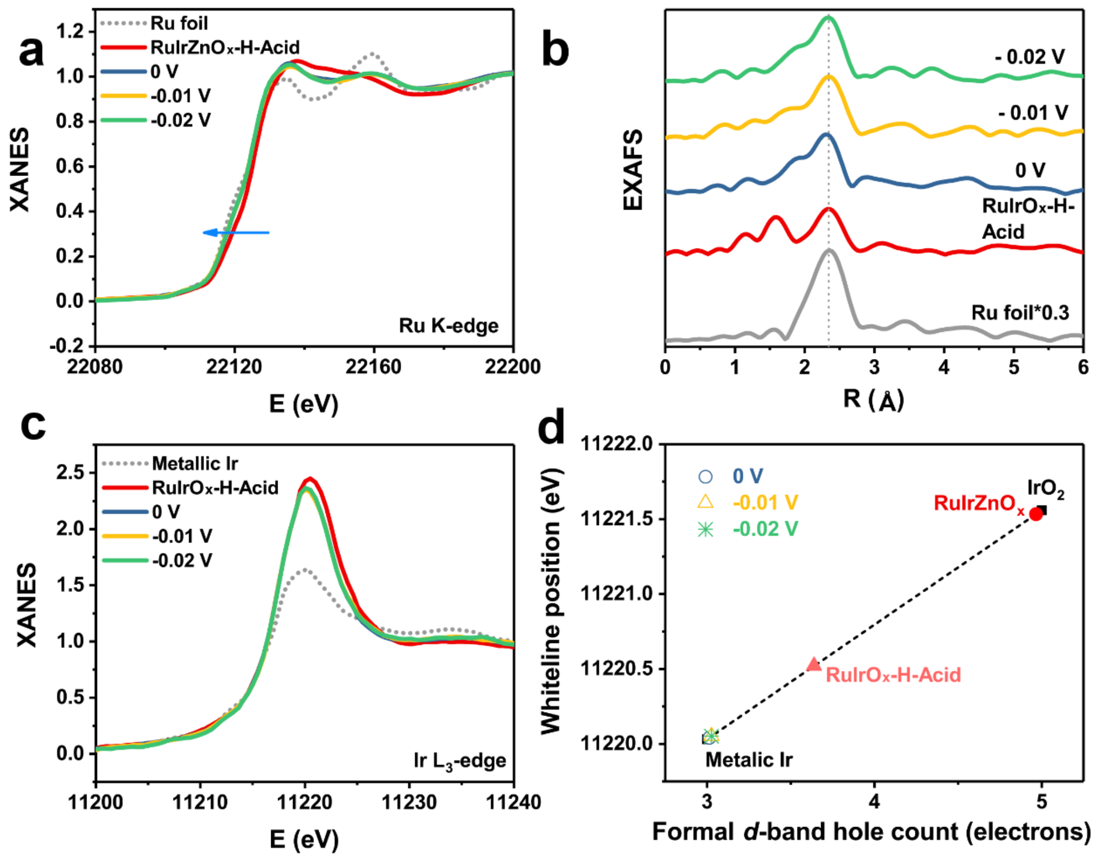

Supplementary Figure 32. operando XAS of RuIrO$_x$ under HER condition in 0.5 M H_2SO_4.

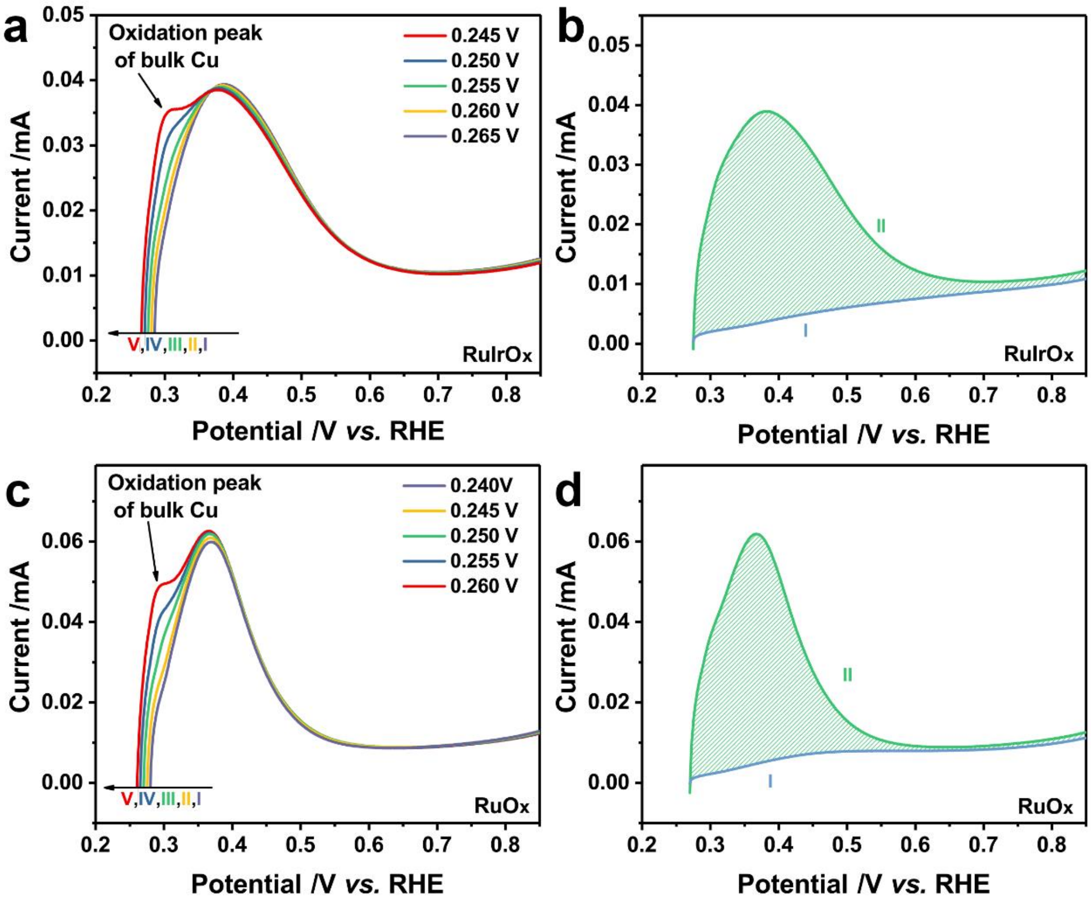

Supplementary Figure 33. Electrochemical surface area (ECSA) measurement of $RuIrO_x$ and RuO_x nano-netcages. Cu-UPD in 0.5 M H_2SO_4 in the presence (I-V) of 5 mM $CuSO_4$ on $RuIrO_x$ (**a**) and RuO_x (**c**). Cu-UPD in 0.5 M H_2SO_4 in the absence (I) and presence (II) of 5 mM $CuSO_4$ on $RuIrO_x$ (**b**) and RuO_x (**d**).

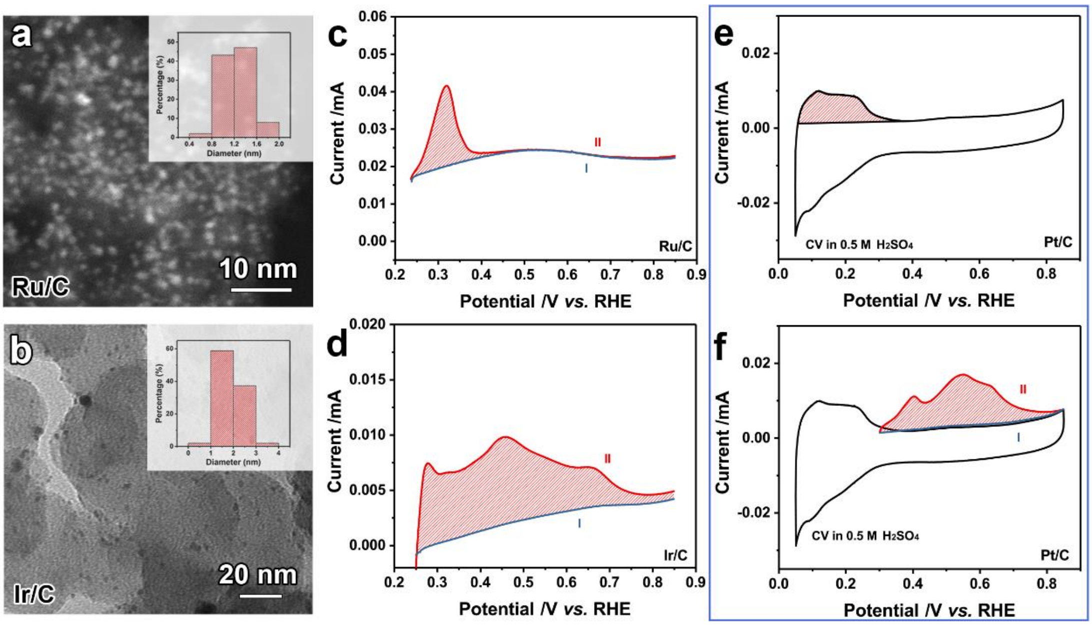

Supplementary Figure 34. Characterization and ECSA measurement of commercial Ru/C, Ir/C and Pt/C catalysts. TEM image and size distribution of commercial Ru/C (D_{av} = 1.2 nm) and Ir/C (D_{av} = 2.0 nm) catalysts (**a, b**). Cu-UPD in 0.5 M H_2SO_4 in the absence (I) and presence (II) of 5 mM $CuSO_4$ on commercial Ru/C (**c**), Ir/C (**d**) and Pt/C (**f**) catalysts. The electrode was polarized at 0.24 V (**c**), 0.25 V (**d**) and 0.30 V (**f**) for 100 s to form the UPD layer for I and II. **e**, H-UPD of Pt/C in 0.5 M H_2SO_4.

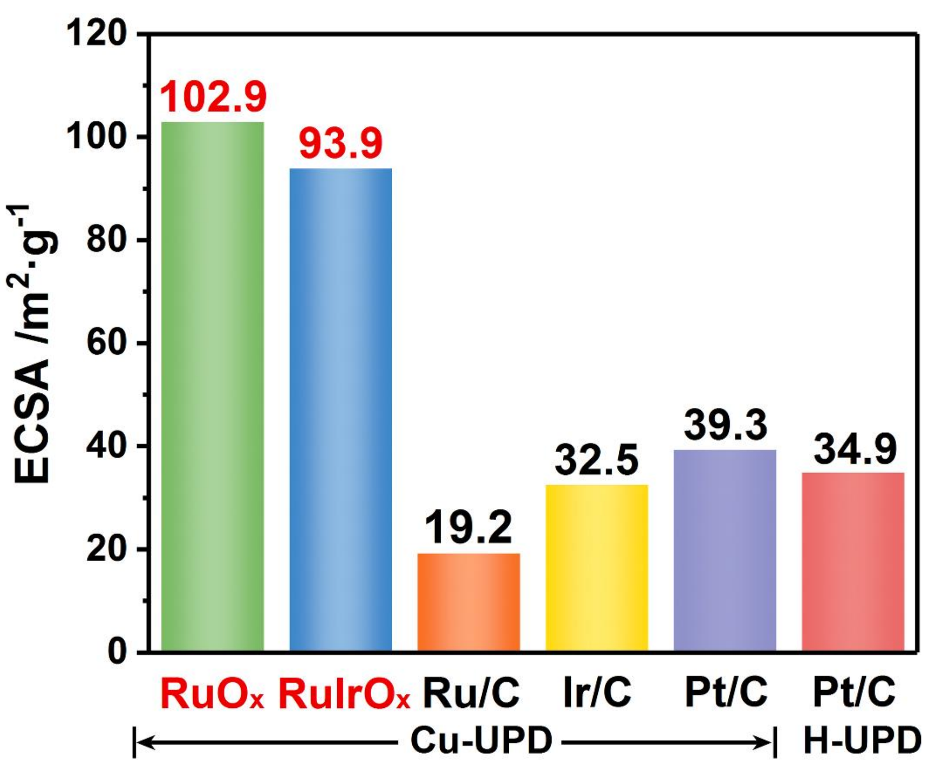

Supplementary Figure 35. Electrochemical surface area (ECSA) of catalysts.

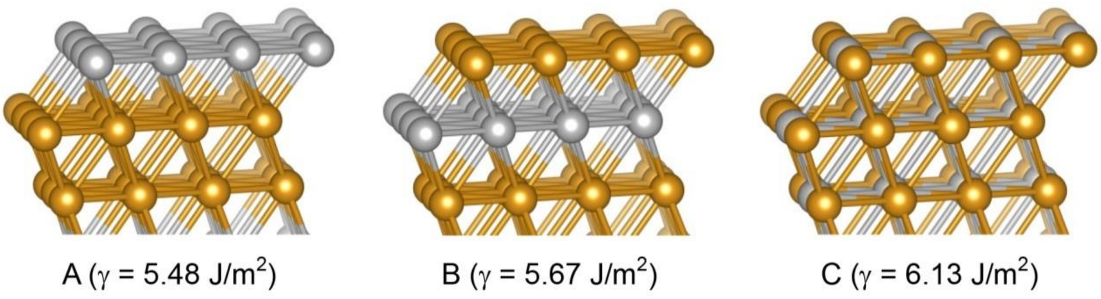

Supplementary Figure 36. Model structures of the *fcc* Ru_2Ir (111) surfaces, with the corresponding surface energies γ given in parentheses.

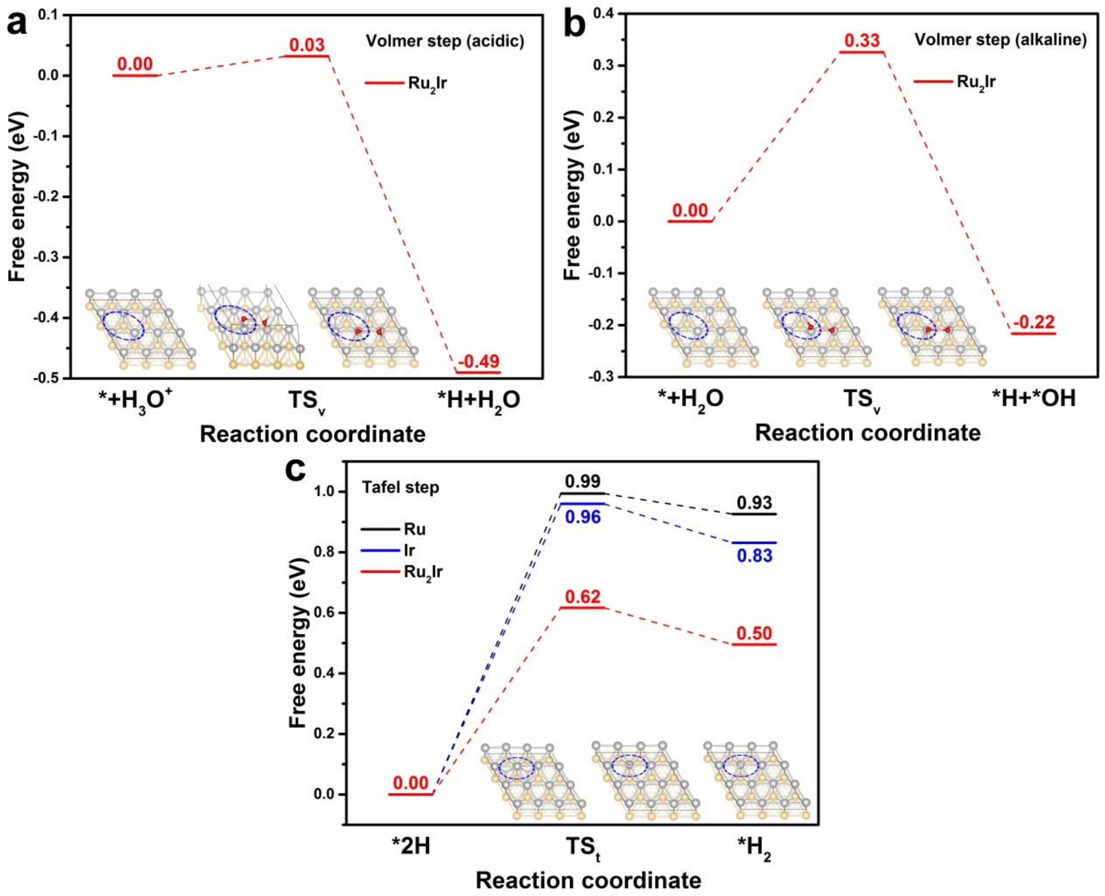

Supplementary Figure 37. The predicted free energy profiles of the elementary steps for HER on Ru (0001) (black line), Ir (111) (blue line) and Ru_2Ir (111) (red line) surfaces under acidic and alkaline conditions. Volmer step under acidic HER condition (**a**). Volmer step under alkaline HER condition (**b**). Tafel step under acidic and alkaline HER conditions (**c**). The O atoms are in red, H in pink, Ir in gray and Ru atoms in gold.

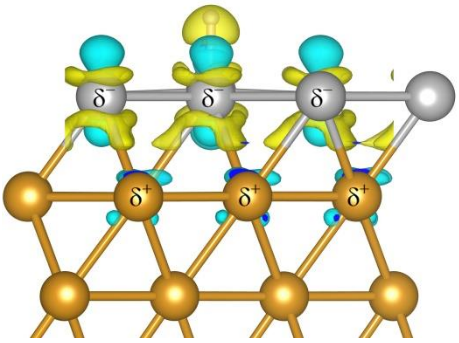

Supplementary Figure 38. Electron density difference of Ru_2Ir with *H. The increase of electron density is indicated by the yellow color, while the decrease of electron density by the cyan color.

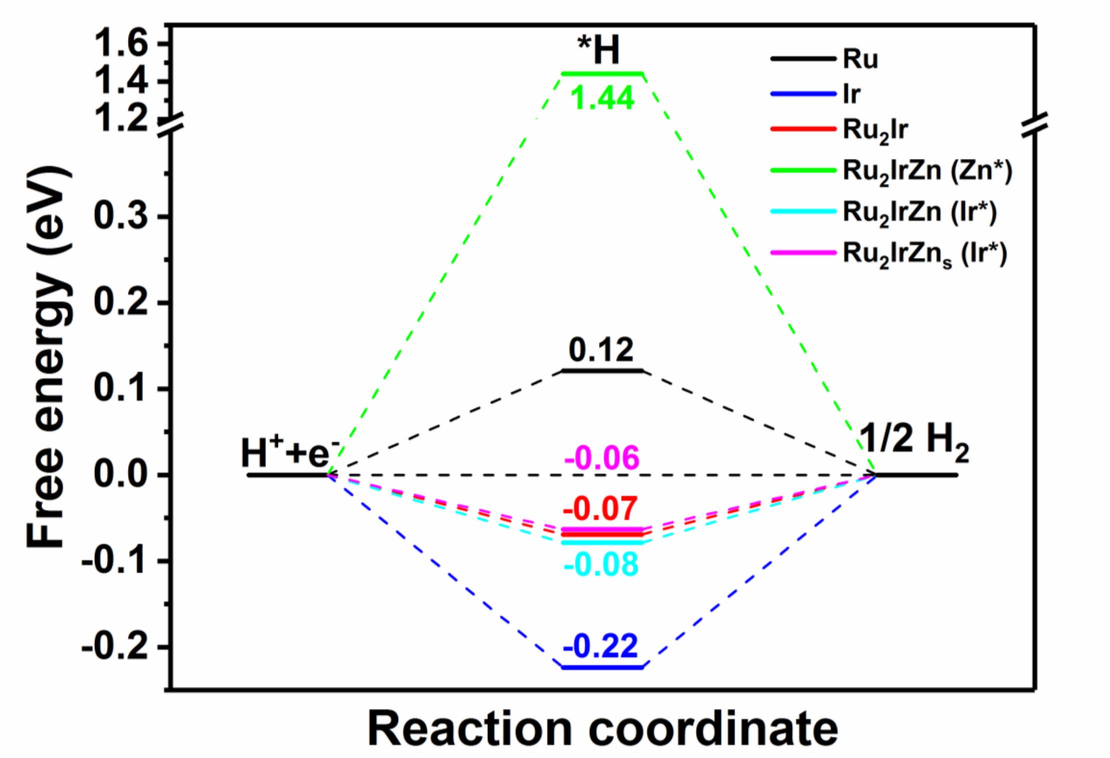

Supplementary Figure 39. The predicted free energy profiles for HER on Ru (0001) surface (black line), Ir (111) surface (blue line), Ru_2Ir (111) surface (red line), Ru_2IrZn (111) surfaces (Zn located at the surface) with Zn (green line) and Ir (cyan line) active sites, and Ru_2IrZn_s (111) surface (Zn incorporated in the slab) with Ir active site (magenta line) at U = 0 eV.

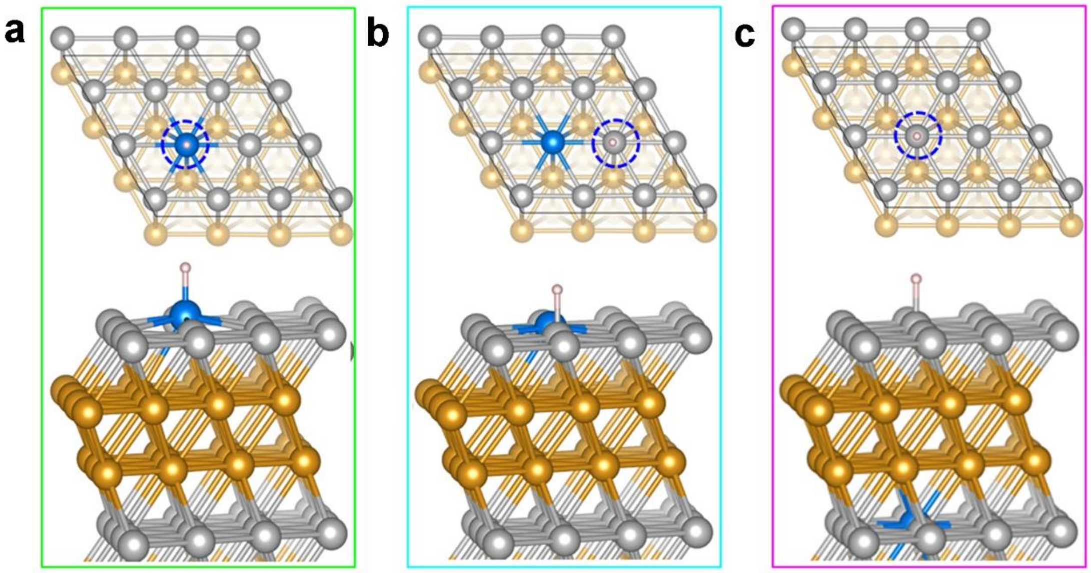

Supplementary Figure 40. Top and side views of the intermediates in HER on Ru_2IrZn (111) surfaces with (a) Zn (green line), (b) Ir (cyan line) active sites, and (c) Ru_2IrZn_s (111) surface with Ir active site (magenta line). The H atoms are in pink, Ir in gray, Ru in gold and Zn atoms in blue.

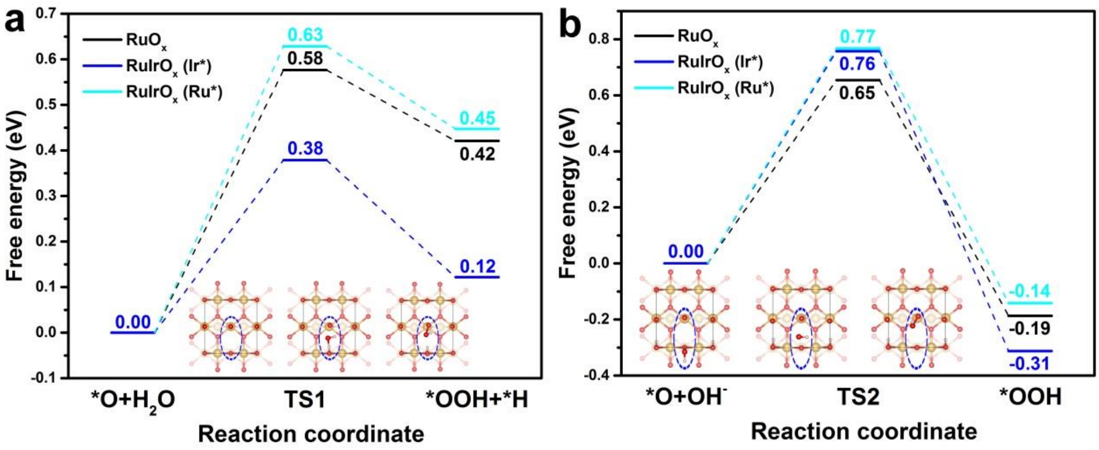

Supplementary Figure 41. The predicted free energy profiles of the RDS for OER on RuO_x (110) surface (black line), $RuIrO_x$ (110) surfaces with Ir (blue line) and Ru (cyan line) as the active sites under acidic (a) and alkaline (b) conditions. The O atoms are in red, H in pink and Ru atoms in gold.

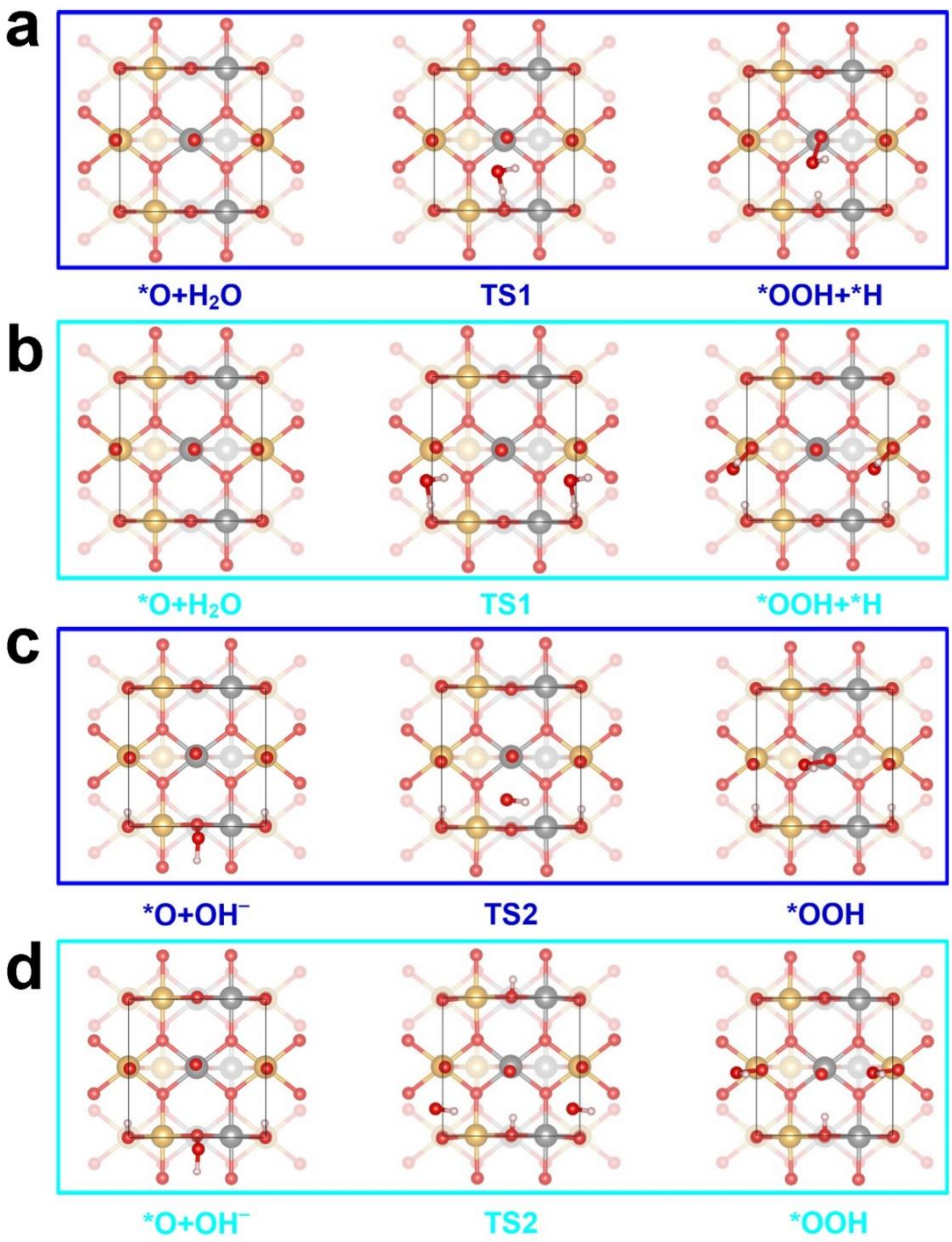

Supplementary Figure 42. Structures of the intermediates and transition states (TS1, TS2) of the RDS for OER on RuIrO*x* (110) surfaces with Ir (blue frame) active sites under acidic condition (a), Ru (cyan frame) active sites under acidic condition (b), Ir (blue frame) active sites under alkaline condition (c) and Ru (cyan frame) active sites under alkaline condition. The O atoms are in red, H in pink, Ir in gray and Ru atoms in gold.

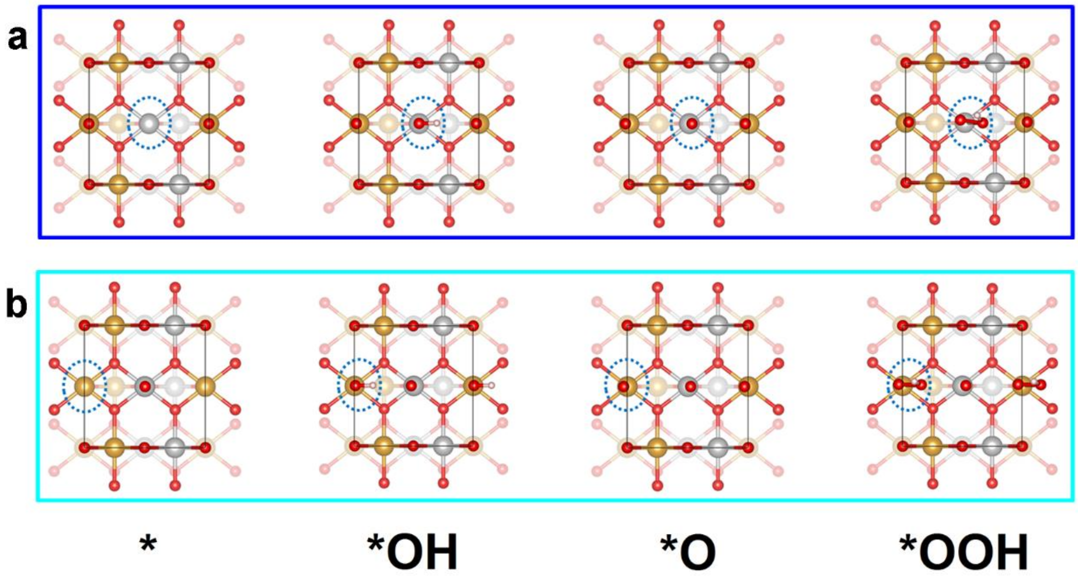

Supplementary Figure 43. Structures of the intermediates in OER on RuIrO$_x$ (110) surfaces with (a) Ir (blue frame) and (b) Ru (cyan frame) active sites. The O atoms are in red, H in pink, Ir in gray and Ru atoms in gold.

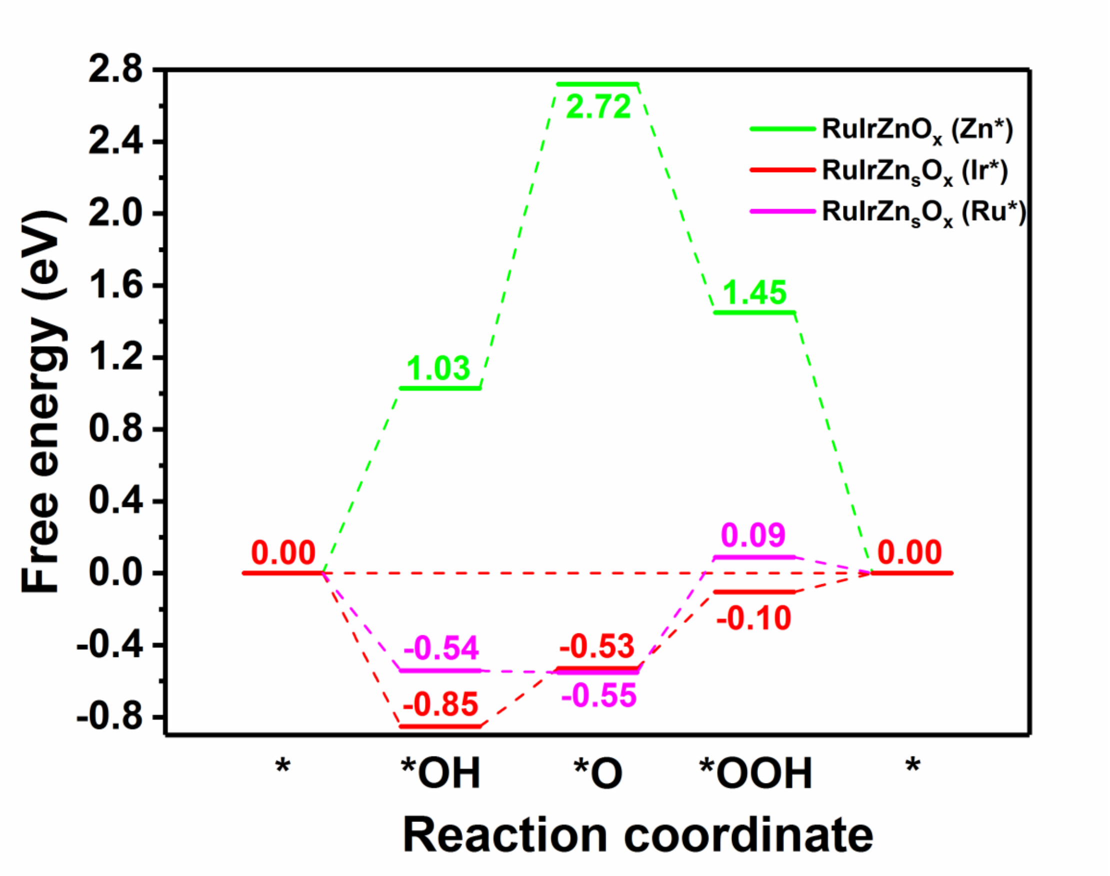

Supplementary Figure 44. The predicted free energy profiles for OER on RuIrZnO$_x$ (110) surface (Zn located at the surface) with Zn active site (green line) and RuIrZn$_s$O$_x$ (110) surfaces (Zn incorporated in the slab) with Ir (red line) and Ru (magenta line) active sites at U = 1.23 eV. The energy uptake of the PLS is 0.43 eV at the Ir site of RuIrZn$_s$O$_x$, even higher than that (0.40 eV) of RuIrO$_x$. OER on the Zn site at the surface is also hindered due to large energy barriers of the first two steps (1.03 eV, 1.69 eV).

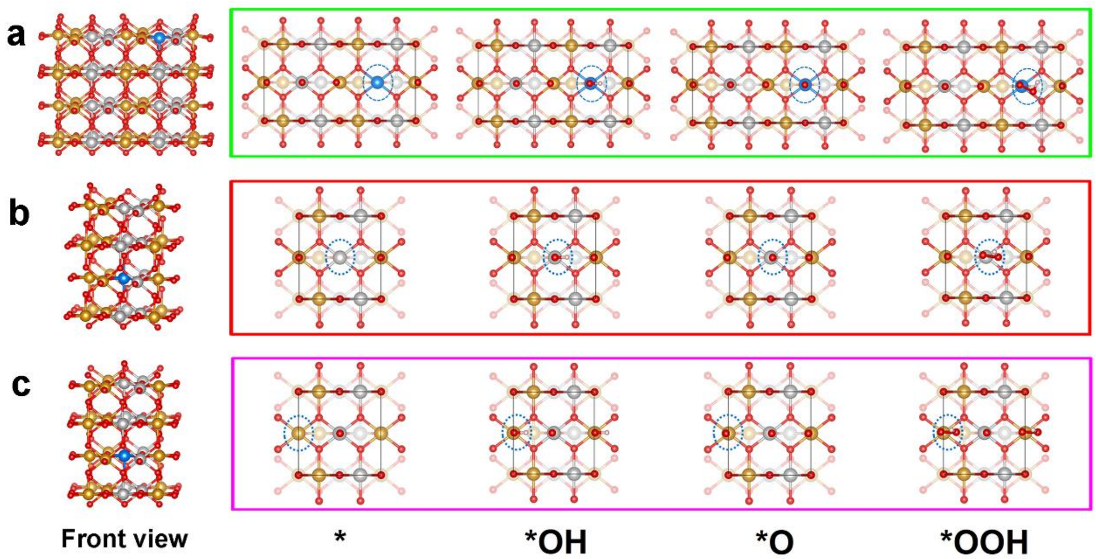

Supplementary Figure 45. Structures of the intermediates in OER on (a) $RuIrZnO_x$ (110) surface with Zn active site (green frame), (b) $RuIrZn_sO_x$ (110) surface with Ir active site (red frame) and (c) $RuIrZn_sO_x$ (110) surface with Ru active site (magenta frame). The O atoms are in red, H in pink, Ir in gray, Ru in gold and Zn atoms in blue.

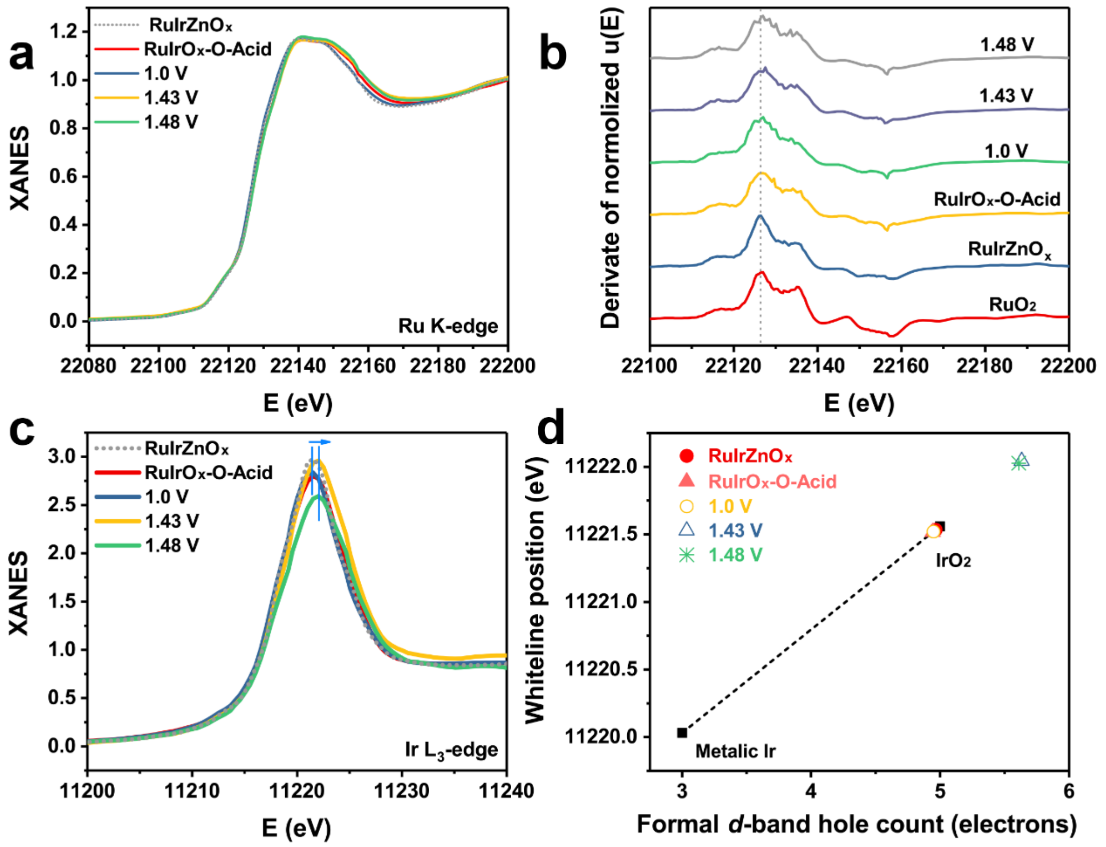

Supplementary Figure 46. operando XAS of RuIrO*x* under OER condition in 0.5 M H_2SO_4.

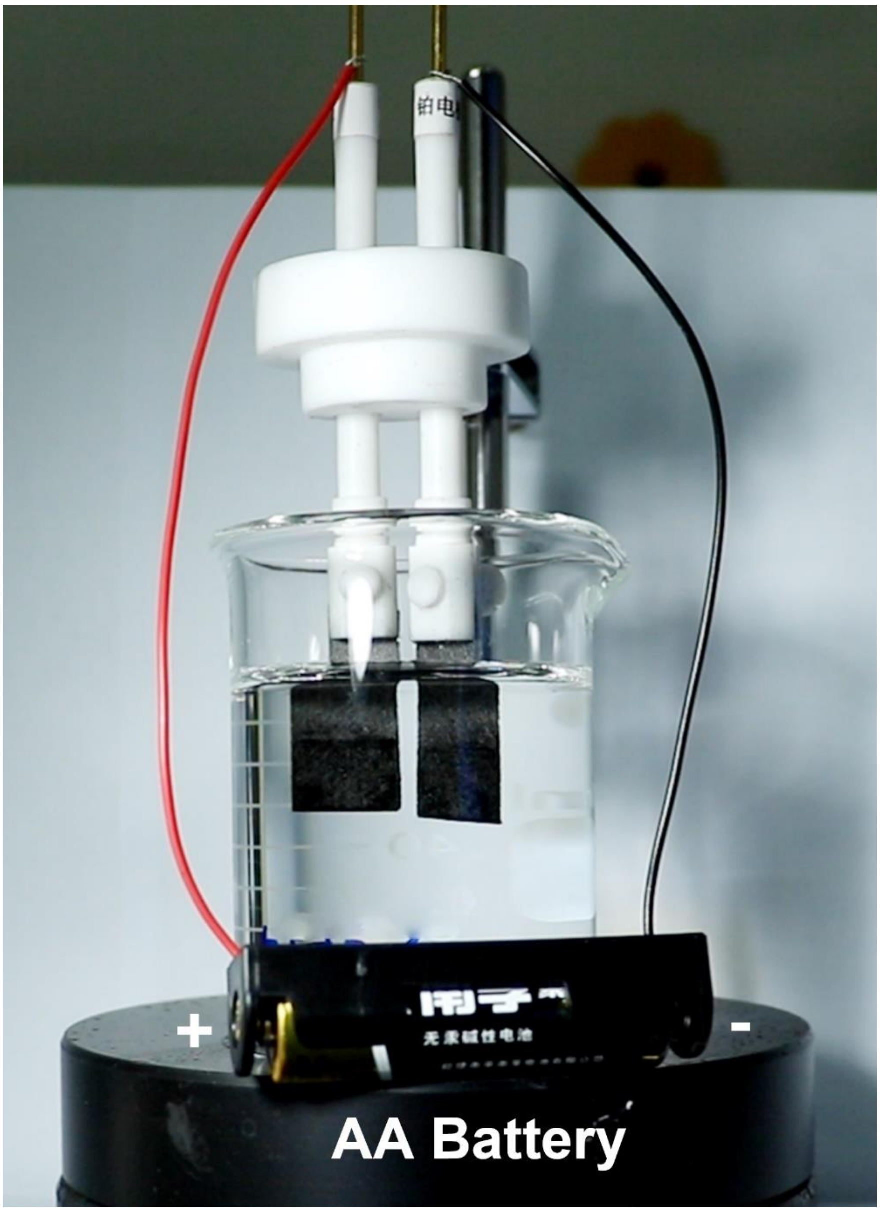

Supplementary Figure 47. Experimental setup with an AA battery for overall water splitting.

Supplementary Tables

Supplementary Table 1. Samples with different Ru:Ir ratios.

Sample	Introduction amount of Ru precursor (μmol)	Introduction amount of Ir precursor (μmol)	Ru:Ir (molar ratio, ICP-OES)
$RuZnO_x$	75	0	1:0
$RuIrZnO_x$-25	62.5	25	1:0.34
$RuIrZnO_x$-50	62.5	50	1:0.46
$RuIrZnO_x$-72.5	62.5	72.5	1:0.47

Supplementary Table 2. Summary of some recently reported catalysts for HER in alkaline electrolyte.

Catalyst	Electrolyte	Catalyst loading amount /mg·cm^{-2}	η (10 mA·cm^{-2}) /mV	Tafel slope /mV·dec^{-1}	Ref.
**$RuIrO_x$**	**1.0 M KOH**	**0.01 (Ru+Ir)**	**13**	**23**	**This work**
Ru@C_2N	1.0 M KOH	0.285	17	38	1
RuCo@NC	1.0 M KOH	0.275	28	31	2
Ni@Ni_2P-Ru	1.0 M KOH	0.283	31	41	3
Pt_3Ni_3	1.0 M KOH	0.015 (Pt)	40	--	4
Au-Ru-2 NWs	1.0 M KOH	0.08	50	31	5
RuP_2 @NPC	1.0 M KOH	1.0	52	69	6
Ru/C_3N_4/C	0.1 M KOH	0.2	79	--	7
Pt/SL-$Ni(OH)_2$	1.0 M KOH	0.016 (Pt)	86 (4 mA·cm^{-2})	--	8
Pt@PCM	1.0 M KOH	--	139	74	9
CoMoSx	0.1 M KOH	0.05	158 (5 mA·cm^{-2})	--	10
Co(OH)2/Pt(111)	0.1 M KOH	--	~248	--	11
MoCx nano-octahedrons	1.0 M KOH	0.8	151	59	12

Supplementary Table 3. Summary of some recently reported catalysts for HER in acidic electrolyte.

Catalyst	Electrolyte	Catalyst loading amount /mg·cm^{-2}	η (10 mA·cm^{-2}) /mV	Tafel slope /mV·dec^{-1}	Ref.
**$RuIrO_x$**	**0.5 M H_2SO_4**	**0.01 (Ru+Ir)**	**12**	**21**	**This work**
Ru@C_2N	0.5 M H_2SO_4	0.285	22	30	1
IrCo@CN	0.5 M H_2SO_4	--	24	23	13
A-Ni–C	0.5 M H_2SO_4	0.283	34	41	14
Ni@Ni_2P-Ru	0.5 M H_2SO_4	0.283	38	31	3
RuP_2 @NPC	0.5 M H_2SO_4	1.0	38	38	6
CoPS nanoplate	0.5 M H_2SO_4	--	48	57	15
Pt-MoS_2	0.5 M H_2SO_4	0.075 (Pt)	53	40	16
$WO_{2.9}$	0.5 M H_2SO_4	0.285	70	50	17
Pt ML Ag NF/Ni foam	0.5 M H_2SO_4	--	70	53	18
Pt NWs/SL-$Ni(OH)_2$	0.5 M H_2SO_4	--	95 (5 mA·cm^{-2})	--	8
SV-MoS_2	0.5 M H_2SO_4	--	170	60	19
$[Mo_3S_{13}]^{2-}$ clusters	0.5 M H_2SO_4	0.1	180	40	20
$CoMoS_x$	0.5 M H_2SO_4	0.05	207 (5 mA·cm^{-2})	--	10
Mesoporous MoS_2	0.5 M H_2SO_4	0.06	233	50	21
Exfoliated WS_2 nanosheets	0.5 M H_2SO_4	0.0065	234	55	22

Supplementary Table 4. The Ru:Ir molar ratio of different samples.

Ru:Ir molar ratio	$RuIrZnO_x$	$RuIrO_x$-H-Alkaline	$RuIrO_x$-H-Acid	$RuIrO_x$-O-Alkaline	$RuIrO_x$-O-Acid
ICP-OES	2.13	2.01	2.18	1.88	1.90

Supplementary Table 5. The calculation of ratio of effective surface area.

	Ru/C	Ir/C	RuO_x	$RuIrO_x$
R (nm)	0.6	1.0	1.5	1.5
Theoretical specific surface area ($m^2 \cdot g^{-1}$)	650.80	136.92	173.55	133.44
ECSA obtained via Cu-UPD ($m^2 \cdot g^{-1}$)	19.2	32.5	102.9	93.9
Ratio of effective surface area (%)	3.0	23.7	59.3	70.4

Supplementary Table 6. Bader charges of atoms in the H adsorbed systems.

Atom [a]	Ru	Ir	Ru_2Ir
Ru*	0.05		
Ir*		0.05	-0.05
Ru_1	-0.03		
Ir_1		-0.04	-0.17
Ru_2	0.07		0.20
Ir_2		0.06	
H	-0.24	-0.12	-0.15

[a] M_1 (M = Ru, Ir) corresponds to the average charge of M atoms in the first layer while M_2 for the second layer.

Supplementary Table 7. Summary of some recently reported catalysts for overall water splitting.

Catalyst	Electrolyte	Cell voltage (10 $mA\cdot cm^{-2}$) /mV	Ref.
**$RuIrO_x$**	**1.0 M KOH**	**1.47**	**This work**
$NiCeO_x$-Au	6.0 M KOH	1.5	23
$NiFeO_x$	1.0 M KOH	1.51	24
Porous MoO_2/NF	1.0 M KOH	1.53	25
NiFe-MOF/NF	1.0 M KOH	1.55	26
MoS_2/Ni_3S_2	1.0 M KOH	1.56	27
NiSe/NF	1.0 M KOH	1.63	28
CoP/NCNHP	1.0 M KOH	1.64	29
$NiCo_2O_4$	1.0 M KOH	1.65	30
$Ni_3ZnC_{0.7}$-550/NF	1.0 M KOH	1.65	31
Ni_5P_4	1.0 M KOH	1.7	32
Ni_3S_2/NF	1.0 M KOH	1.7	33
**$RuIrO_x$**	**0.5 M H_2SO_4**	**1.45**	**This work**
Ir/GF	0.5 M H_2SO_4	1.55	34
IrCoNi	0.5 M H_2SO_4	1.56	35
Ir WNWs	0.1 M $HClO_4$	1.62	36
$IrNi_{0.57}Fe_{0.82}$	0.5 M $HClO_4$	1.64	37
ONPPGC/OCC	0.5 M H_2SO_4	1.75 (5 $mA\cdot cm^{-2}$)	38

Supplementary Notes

Supplementary Note 1. For clarity, we denoted the catalysts under different conditions with different names. Here, "electrochemical activation" indicates running for several CV cycles within the potential range of specific electrochemical reactions.

RuIrO$_x$-H-Alkaline: The catalyst after electrochemical activation under alkaline HER.

RuIrO$_x$-H-Acid: The catalyst after electrochemical activation under acidic HER.

RuIrO$_x$-O-Alkaline: The catalyst after electrochemical activation under alkaline OER.

RuIrO$_x$-O-Acid: The catalyst after electrochemical activation under acidic OER.

We can find that the X-ray counts of Zn and O elements is greatly decreased after the HER reaction. After OER reaction, the X-ray counts of Zn element is vastly reduced, while the counts of O are not significantly decreased (Supplementary Fig. 22). The EDX mapping results are consistent with the line-scan profiles (Supplementary Fig. 23). As shown in the SEM images (Supplementary Fig. 24), after electrochemical activation, the *in-situ* formed RuIrO$_x$ still retain the morphology of hollow nanobox, with abundant pores on the walls, which is in good agreement with the TEM results.

XPS results (Supplementary Fig 28) show that, compared to the pre-catalyst RuIrZnO$_x$, for the catalysts after electrochemical activation under alkaline/acidic HER (RuIrO$_x$-H-Alkaline, RuIrO$_x$-H-Acid), the Ru 3p peaks shifted by 0.4 eV to lower binding energy, suggesting a lower electron density at the Ru sites. Deconvolution of Ru 3p$_{3/2}$ show that the peak for elemental Ru (at 461.1 eV) emerged after HER[39]. Similarly, the Ir 4f peaks for RuIrO$_x$-H-Alkaline and RuIrO$_x$-H-Acid also shifted to lower binding energy, and the peak for elemental Ir 4f$_{7/2}$ (at 60.9 eV) emerged after HER[40]. These results prove that during HER, Ru/Ir would undergo *in-situ* reduction and form RuIr alloy, which serves as the real active species for HER. In contrast, for the catalysts after OER activation (RuIrO$_x$-O-Alkaline, RuIrO$_x$-O-Acid), the Ru 3p and Ir 4f spectrum do not show significant changes with respect to those for the pre-catalyst,

indicating that after OER, Ru/Ir still exists in the form of oxides, and retains a good stability during OER process.

The position of XANES absorption threshold for Ru K-edge can reflect the electronic structural information. We found that after HER activation, the absorption thresholds for Ru in $RuIrO_x$-H-Alkaline and $RuIrO_x$-H-Acid shifted to lower energy, approaching that for Ru foil, indicating that the oxidation state of Ru in $RuIrO_x$-H-Alkaline and $RuIrO_x$-H-Acid decreased after HER (Supplementary Fig. 29). In the corresponding EXAFS, a new peak emerged at 2.35 Å for $RuIrO_x$-H-Alkaline and $RuIrO_x$-H-Acid, corresponding to the scattering of Ru-Ru/Ir bonds. The white line position of Ir can reflect the formal *d*-band hole count which is related to the oxidation state of Ir[41-43]. Similarly, after HER activation, the white line position of $RuIrO_x$-H-Alkaline and $RuIrO_x$-H-Acid shifted to lower energy, approaching that for metallic Ir. Also, a new peak corresponding to Ir-Ru/Ir bond emerged in the EXFAS. These results prove that during HER, Ru/Ir would undergo *in-situ* reduction. Similar to the XPS results, the XANES and EXAFS data for $RuIrO_x$-O-Alkaline and $RuIrO_x$-O-Acid are quite similar to that for the pre-catalyst, indicating that after OER, Ru/Ir still exists in the form of oxides, with the electronic structures and coordination environments barely altered, and the species remains stable during the OER process.

The quantitative coordination configuration of Ru and Ir atoms in $RuIrO_x$ nano-netcages after electrochemical activation can be obtained by EXAFS fitting, as shown in Supplementary Fig.30. After HER activation, the Ru-Ru/Ir and Ir-Ir/Ru scattering pair emerged, which indicated that the Ru/Ir oxides would undergo *in-situ* reduction and form RuIr alloy. After OER activation, the coordination number and the average bond length of Ru-O bond and Ir-O bond of $RuIrO_x$-O-Alkaline and $RuIrO_x$-O-Acid remain close to the $RuIrZnO_x$ pre-catalyst, suggesting the essentially identical coordination environment of Ru and Ir atoms before and after OER activation.

Supplementary Note 2. Supplementary Fig. 31d shows the white line position of the catalysts at different applied potentials as a function of the formal *d*-band hole count to reveal the oxidation states of Ir species[41-43]. The results indicated that, the number of

d-band holes in $RuIrZnO_x$ pre-catalyst is close to IrO_2, indicating the near +4 oxidation state which was consistence with the XPS results. And the number of d-band holes significantly decrease after HER electrochemical activation. Under HER potentials, the formal d-band hole count of Ir in our sample was almost identical to that of metallic Ir, implying a similar oxidation state.

As for acidic HER (Supplementary Fig.32), $RuIrO_x$-H-Acid was also converted into Ru_2Ir alloy (with a Ru:Ir molar ration as 2.18 of $RuIrO_x$-H-Acid), displaying HER activity.

Supplementary Note 3. In order to further verify the structural advantages of the hollow nano-netcage architecture, we assessed the theoretical specific surface areas of the samples in two different morphologies: (1) nanoparticles, (2) nano-netcages. The nanoparticles are assumed to take a spherical shape; as for the nano-netcage made of interconnecting ultrathin nanowires, we calculated the specific surface area of the nanowires. The calculations are listed below:

(1) Nanoparticles

The radius of a nanoparticle: R

The surface area of a nanoparticle: $A = 4\pi R^2$

The volume of a nanoparticle: $V = \frac{4}{3}\pi R^3$

The volume of a single unit cell: V_c

The number of metal atoms in a unit cell: n

The number of metal atoms in a nanoparticle: $n * \frac{V}{V_C} = \frac{\frac{4}{3}\pi R^3}{V_C} = \frac{4\pi n R^3}{3V_C}$

The mass of a nanoparticle: $m = n * \frac{V}{V_C} \cdot \frac{M}{N_A} = \frac{4\pi n R^3}{3V_C} \cdot \frac{M}{N_A}$

The specific surface area of a nanoparticle: $\frac{A}{m} = \frac{4\pi R^2}{\frac{4\pi n R^3}{3V_C} \cdot \frac{M}{N_A}} = \frac{3V_C N_A}{nRM}$

(2) Nano-netcage:

The radius of a nanowire: R

The length of a nanowire: x

The surface area of a nanowire: $A = 2\pi Rx$

The volume of a nanowire: $V = \pi x R^2$

The volume of a unit cell: V_c

The number of metal atoms in a unit cell: n

The number of metal atoms in a nanowire: $n * \frac{V}{V_c} = \frac{\pi n x R^2}{V_c}$

The mass of a nanowire: $m = \frac{\pi n x R^2}{V_c} \cdot \frac{M}{N_A}$

The specific surface area of a nanowire: $\frac{A}{m} = \frac{2\pi Rx}{\frac{\pi n x R^2}{V_c} \cdot \frac{M}{N_A}} = \frac{2V_C N_A}{nRM}$

Supplementary Note 4. The Tafel slope by $RuIrO_x$ in H_2SO_4 solution (21 $mV \cdot dec^{-1}$) is similar to that in KOH solution (23 $mV \cdot dec^{-1}$), indicating that both the acidic and alkaline HER follow the Volmer-Tafel mechanism. As shown in Supplementary Fig. 37, the Tafel step has significantly higher free energy barrier than the Volmer step under both acidic and alkaline conditions, confirming our previous assumption from experiments. Thus, the hydrogen adsorption is a valid activity descriptor for HER in our work. Note that there is a remarkable decrease of free energy barrier of the Tafel step on the Ru_2Ir system compared with pure Ru or Ir, which is consistent with the experiment observation.

Supplementary Note 5. The Tafel slopes of OER on $RuIrO_x$ are 42 $mV \cdot dec^{-1}$ in H_2SO_4 solution and 50 $mV \cdot dec^{-1}$ in KOH solution, suggesting that the rate determining step under both acidic and alkaline conditions is the third elementary step. Meanwhile, the calculated energy profile (Figure 4b) for OER also reveals that the potential limiting step (PLS) is the third step from *O to *OOH, in good agreement with the experimental observation. Water dissociation and the formation of *OOH intermediate in the third

step are studied in detail, as shown in Supplementary Fig. 40 and 41. The Ir site of $RuIrO_x$ possesses the lowest activation energy (0.38 eV) and contributes to better performance than pure RuO_x under acidic condition.

Note that the kinetic investigations of *OOH formation indicates that the water dissociation process ($*O + H_2O \rightarrow *OOH + H^+ + e^-$) is kinetically more favorable than OER with hydroxide anion ($*O + OH^- \rightarrow *OOH + e^-$) under alkaline conditions. Thus, we conclude that the OER proceeds with water molecules as reactants even under alkaline conditions, and this is also supported by the fact that the amount of H_2O (56 mol/L) is much larger than that of OH^- even in high-concentration alkaline solutions. The Ir site of $RuIrO_x$ has stronger interactions with OOH, leading to a remarkable decrease in the free energy barrier for the PLS. Thus, the excellent OER activity can be attributed to a high OOH binding energy here.

Supplementary References.

1. Mahmood, J. *et al.* An efficient and pH-universal ruthenium-based catalyst for the hydrogen evolution reaction. *Nat. Nanotechnol.* **12**, 441-446 (2017).
2. Su, J. *et al.* Ruthenium-cobalt nanoalloys encapsulated in nitrogen-doped graphene as active electrocatalysts for producing hydrogen in alkaline media. *Nat. Commun.* **8**, 14969 (2017).
3. Liu, Y. *et al.* Ru Modulation Effects in the Synthesis of Unique Rod-like Ni@Ni_2P-Ru Heterostructures and Their Remarkable Electrocatalytic Hydrogen Evolution Performance. *J. Am. Chem. Soc.* **140**, 2731-2734 (2018).
4. Wang, P., Jiang, K., Wang, G., Yao, J. & Huang, X. Phase and Interface Engineering of Platinum-Nickel Nanowires for Efficient Electrochemical Hydrogen Evolution. *Angew. Chem. Int. Ed.* **55**, 12859-12863 (2016).
5. Lu, Q. *et al.* Crystal phase-based epitaxial growth of hybrid noble metal nanostructures on 4H/fcc Au nanowires. *Nat. Chem.* **10**, 456-461 (2018).
6. Pu, Z., Amiinu, I.S., Kou, Z., Li, W. & Mu, S. RuP_2 -Based Catalysts with Platinum-like Activity and Higher Durability for the Hydrogen Evolution Reaction at All pH Values. *Angew. Chem. Int Ed.* **56**, 11559-11564 (2017).
7. Zheng, Y. *et al.* High Electrocatalytic Hydrogen Evolution Activity of an Anomalous Ruthenium Catalyst. *J. Am. Chem. Soc.* **138**, 16174-16181 (2016).
8. Yin, H. *et al.* Ultrathin platinum nanowires grown on single-layered nickel hydroxide with high hydrogen evolution activity. *Nat. Commun.* **6**, 6430 (2015).
9. Zhang, H. *et al.* Dynamic traction of lattice-confined platinum atoms into mesoporous carbon matrix for hydrogen evolution reaction. *Sci. Adv.* **4**, eaao6557 (2018)
10. Staszak-Jirkovsky, J. *et al.* Design of active and stable Co-Mo-S_x chalcogels as pH-universal catalysts for the hydrogen evolution reaction. *Nat. Mater.* **15**, 197-203 (2016).
11. Subbaraman, R. *et al.* Trends in activity for the water electrolyser reactions on 3d M(Ni,Co,Fe,Mn) hydr(oxy)oxide catalysts. *Nat. Mater.* **11**, 550-557 (2012).

12. Wu, H.B., Xia, B.Y., Yu, L., Yu, X.Y. & Lou, X.W. Porous molybdenum carbide nano-octahedrons synthesized via confined carburization in metal-organic frameworks for efficient hydrogen production. *Nat. Commun.* **6**, 6512 (2015).

13. Jiang, P. *et al.* Tuning the Activity of Carbon for Electrocatalytic Hydrogen Evolution via an Iridium-Cobalt Alloy Core Encapsulated in Nitrogen-Doped Carbon Cages. *Adv. Mater.* **30**, 1705324 (2018).

14. Fan, L. *et al.* Atomically isolated nickel species anchored on graphitized carbon for efficient hydrogen evolution electrocatalysis. *Nat. Commun.* **7**, 10667 (2016).

15. Caban-Acevedo, M. *et al.* Efficient hydrogen evolution catalysis using ternary pyrite-type cobalt phosphosulphide. *Nat. Mater.* **14**, 1245-1251 (2015).

16. Huang, X. *et al.* Solution-phase epitaxial growth of noble metal nanostructures on dispersible single-layer molybdenum disulfide nanosheets. *Nat. Commun.* **4**, 1444 (2013).

17. Li, Y.H. *et al.* Local atomic structure modulations activate metal oxide as electrocatalyst for hydrogen evolution in acidic water. *Nat. Commun.* **6**, 8064 (2015).

18. Li, M. et al. Pt monolayer coating on complex network substrate with high catalytic activity for the hydrogen evolution reaction. *Sci. Adv.* **4**, e1400268 (2015)

19. Li, H. *et al.* Corrigendum: Activating and optimizing MoS_2 basal planes for hydrogen evolution through the formation of strained sulphur vacancies. *Nat. Mater.* **15**, 364 (2016).

20. Kibsgaard, J., Jaramillo, T.F. & Besenbacher, F. Building an appropriate active-site motif into a hydrogen-evolution catalyst with thiomolybdate $[Mo_3S_{13}]^{2-}$ clusters. *Nat. Chem.* **6**, 248-253 (2014).

21. Kibsgaard, J., Chen, Z., Reinecke, B.N. & Jaramillo, T.F. Engineering the surface structure of MoS_2 to preferentially expose active edge sites for electrocatalysis. *Nat. Mater.* **11**, 963-969 (2012).

22. Voiry, D. *et al.* Enhanced catalytic activity in strained chemically exfoliated WS_2 nanosheets for hydrogen evolution. *Nat. Mater.* **12**, 850-855 (2013).

23. Ng, J.W.D. *et al.* Gold-supported cerium-doped NiO_x catalysts for water oxidation. *Nature Energy* **1** (2016).
24. Wang, H. *et al.* Bifunctional non-noble metal oxide nanoparticle electrocatalysts through lithium-induced conversion for overall water splitting. *Nat. Commun.* **6**, 7261 (2015).
25. Jin, Y. *et al.* Porous MoO_2 Nanosheets as Non-noble Bifunctional Electrocatalysts for Overall Water Splitting. *Adv. Mater.* **28**, 3785-3790 (2016).
26. Duan, J., Chen, S. & Zhao, C. Ultrathin metal-organic framework array for efficient electrocatalytic water splitting. *Nat. Commun.* **8**, 15341 (2017).
27. Zhang, J. *et al.* Interface Engineering of MoS_2 /Ni_3S_2 Heterostructures for Highly Enhanced Electrochemical Overall-Water-Splitting Activity. *Angew. Chem. Int. Ed.* **55**, 6702-6707 (2016).
28. Tang, C., Cheng, N., Pu, Z., Xing, W. & Sun, X. NiSe Nanowire Film Supported on Nickel Foam: An Efficient and Stable 3D Bifunctional Electrode for Full Water Splitting. *Angew. Chem. Int. Ed.* **54**, 9351-9355 (2015).
29. Pan, Y. *et al.* Core-Shell ZIF-8@ZIF-67-Derived CoP Nanoparticle-Embedded N-Doped Carbon Nanotube Hollow Polyhedron for Efficient Overall Water Splitting. *J. Am. Chem. Soc.* **140**, 2610-2618 (2018).
30. Gao, X. *et al.* Hierarchical $NiCo_2O_4$ Hollow Microcuboids as Bifunctional Electrocatalysts for Overall Water-Splitting. *Angew. Chem. Int. Ed.* **55**, 6290-6294 (2016).
31. Tang, T. *et al.* Electronic and Morphological Dual Modulation of Cobalt Carbonate Hydroxides by Mn Doping toward Highly Efficient and Stable Bifunctional Electrocatalysts for Overall Water Splitting. *J. Am. Chem. Soc.* **139**, 8320-8328 (2017).
32. Ledendecker, M. *et al.* The synthesis of nanostructured Ni_5P_4 films and their use as a non-noble bifunctional electrocatalyst for full water splitting. *Angew. Chem. Int. Ed.* **54**, 12361-12365 (2015).
33. Feng, L.L. *et al.* High-index faceted Ni_3S_2 nanosheet arrays as highly active and ultrastable electrocatalysts for water splitting. *J. Am. Chem. Soc.***137**, 14023-

14026 (2015).

34. Zhang, J. *et al.* Iridium nanoparticles anchored on 3D graphite foam as a bifunctional electrocatalyst for excellent overall water splitting in acidic solution. *Nano Energy* **40**, 27-33 (2017).

35. Feng, J. *et al.* Iridium-Based Multimetallic Porous Hollow Nanocrystals for Efficient Overall-Water-Splitting Catalysis. *Adv. Mater.* **29** (2017).

36. Fu, L., Yang, F., Cheng, G. & Luo, W. Ultrathin Ir nanowires as high-performance electrocatalysts for efficient water splitting in acidic media. *Nanoscale* **10**, 1892-1897 (2018).

37. Fu, L., Cheng, G. & Luo, W. Colloidal synthesis of monodisperse trimetallic IrNiFe nanoparticles as highly active bifunctional electrocatalysts for acidic overall water splitting. *J. Mater. Chem. A* **5**, 24836-24841 (2017).

38. Lai, J. *et al.* Unprecedented metal-free 3D porous carbonaceous electrodes for full water splitting. *Energy. Environ.Sci.* **9**, 1210-1214 (2016).

39. Cheng, C., Shah, S.S.A., Najam, T., Qi, X. & Wei, Z. Improving the electrocatalytic activity for hydrogen evolution reaction by lowering the electrochemical impedance of RuO_2/Ni-P. *Electrochim. Acta* **260**, 358-364 (2018).

40. Weber, D. et al. Trivalent Iridium Oxides: Layered Triangular Lattice Iridate $K_{0.75}Na_{0.25}IrO_2$ and Oxyhydroxide IrOOH. *Chem. Mater.* **29**, 8338-8345 (2017).

41. Mo, Y. et al. In Situ Iridium L_{III}-Edge X-ray absorption and surface enhanced Raman spectroscopy of electrodeposited iridium oxide films in aqueous electrolytes. *J. Phys. Chem. B* **106**, 3681-3686 (2002).

42. Hillman, A.R., Skopek, M.A. & Gurman, S.J. X-Ray spectroscopy of electrochemically deposited iridium oxide films: detection of multiple sites through structural disorder. *Phys. Chem. Chem. Phys.* **13**, 5252-5263 (2011).

43. Nong, H.N. et al. A unique oxygen ligand environment facilitates water oxidation in hole-doped $IrNiO_x$ core–shell electrocatalysts. *Nature Catalysis* **1**, 841-851 (2018).
